# Advanced Nanotheranostics of CRISPR/Cas for Viral Hepatitis and Hepatocellular Carcinoma

**DOI:** 10.1002/advs.202102051

**Published:** 2021-10-19

**Authors:** Huimin Kong, Enguo Ju, Ke Yi, Weiguo Xu, Yeh‐Hsing Lao, Du Cheng, Qi Zhang, Yu Tao, Mingqiang Li, Jianxun Ding

**Affiliations:** ^1^ Laboratory of Biomaterials and Translational Medicine Center for Nanomedicine and Biotherapy Center The Third Affiliated Hospital Sun Yat‐sen University 600 Tianhe Road Guangzhou 510630 P. R. China; ^2^ Key Laboratory of Polymer Ecomaterials Changchun Institute of Applied Chemistry Chinese Academy of Sciences 5625 Renmin Street Changchun 130022 P. R. China; ^3^ Department of Biomedical Engineering Columbia University 3960 Broadway Lasker Room 450 New York NY 10032 USA; ^4^ PCFM Lab of Ministry of Education School of Materials Science and Engineering Sun Yat‐sen University 135 Xingangxi Road Guangzhou 510275 P. R. China; ^5^ Guangdong Provincial Key Laboratory of Liver Disease Research 600 Tianhe Road Guangzhou 510630 P. R. China

**Keywords:** CRISPR/Cas, gene editing, hepatocellular carcinoma, nanotheranostics, viral hepatitis

## Abstract

Liver disease, particularly viral hepatitis and hepatocellular carcinoma (HCC), is a global healthcare burden and leads to more than 2 million deaths per year worldwide. Despite some success in diagnosis and vaccine development, there are still unmet needs to improve diagnostics and therapeutics for viral hepatitis and HCC. The emerging clustered regularly interspaced short palindromic repeat/associated proteins (CRISPR/Cas) technology may open up a unique avenue to tackle these two diseases at the genetic level in a precise manner. Especially, liver is a more accessible organ over others from the delivery point of view, and many advanced strategies applied for nanotheranostics can be adapted in CRISPR‐mediated diagnostics or liver gene editing. In this review, the focus is on these two aspects of viral hepatitis and HCC applications. An overview on CRISPR editor development and current progress in clinical trials is first given, followed by highlighting the recent advances integrating the merits of gene editing and nanotheranostics. The promising systems that are used in other applications but may hold potentials in liver gene editing are also discussed. This review concludes with the perspectives on rationally designing the next‐generation CRISPR approaches and improving the editing performance.

## Graphical abstract

Herein, the clustered regularly interspaced short palindromic repeat (CRISPR) nanotheranostics developed for viral hepatitis and hepatocellular carcinoma are reviewed, and rational design considerations for the next‐generation nanomedicine approaches to advance liver gene editing are discussed, highlighting their significances in clinical applications.

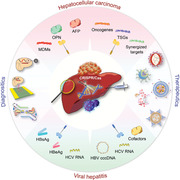

## 1 Introduction

The liver is the metabolism center of the human body, actively involved in many necessary physiological reactions. Its dysfunctions caused by liver‐associated diseases (*e.g*., chronic inflammation and cancer) can lead to detrimental pathophysiological consequences and severely impair the human body's normal functionalities.^[^ ^1^ ^]^ Viral hepatitis, the inflamed liver damage with a high incidence, is caused by viral infections and often compromises the liver functions, which may consequently threaten patient's health. Hepatitis viruses type B (HBV) and type C (HCV) preferentially infect hepatocytes, and chronic HBV and HCV infections lead to many complications, including chronic cirrhosis and liver cancers.^[^ ^2^ ^]^ Hepatocellular carcinoma (HCC), accounting for nearly 90% of liver tumors, is one of the most common fatal cancers in the world.^[^ ^2^ ^]^ Therefore, prevention and confinement of liver‐associated diseases, especially viral hepatitis and HCC, are unmet needs of the field.

Despite some success in serum detection and vaccine development,^[^ ^3^ ^]^ there is still a lack of precise diagnoses and treatments at the genetic level for hepatitis viral infections and HCC. The emerging gene engineering technologies, such as zinc‐finger nucleases (ZFNs), transcription activator‐like effector nucleases (TALENs), and clustered regularly interspaced short palindromic repeat/associated proteins (CRISPR/Cas), may fill the technical gaps.^[^ ^4^ ^]^ Particularly, with its high efficacy and programmable designs, CRISPR/Cas technology is attractive for a broad spectrum of applications for diagnostics and therapeutics.

In the last few years, the CRISPR/Cas technology has stimulated significant efforts in gene editing for various application aspects.^[^ ^5^ ^]^ In the fields of viral hepatitis and HCC, CRISPR/Cas technology has been applied to advance liver theranostics with great accuracy and versatility. Notably, as most delivery carriers, especially non‐viral nanoparticles, accumulate in the liver when given through systemic administration,^[^ ^6^ ^]^ the liver may be the most suitable organ with better editing efficacy outperforming other tissues, which may be easier to meet the clinical goals. For one thing, perceived as a site with the privilege of the immune system, the liver tends to induce immune tolerance instead of immunogenicity.^[^ ^7^ ^]^ For another, many nanoparticles hold liver tropism, which may accumulate CRISPR/Cas cargos in nanoscale for further editing applications.

**Figure 1:**
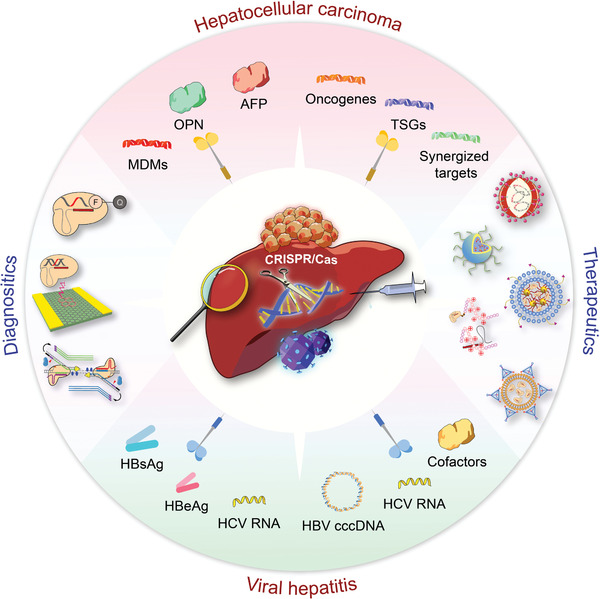
CRISPR/Cas technology for theranostics of viral hepatitis and HCC. This review discusses the emerging CRISPR/Cas toolkits to advance liver‐targeting nanotheranostics, especially for viral hepatitis and HCC. The CRISPR‐based diagnostics part in this review mainly focuses on the advance of new sensing appraoches and discovery of promising markers, while the CRISPR‐based nanomedicine part concentrates on the discussion of potential therapeutic targets and highlights delivery platforms for liver gene editing.

This review overviews various CRISPR/Cas applications and the potentials for viral hepatitis and HCC, followed by our perspectives on how to facilitate the diagnostics and therapeutics of viral hepatitis and HCC by integrating the merits of CRISPR/Cas gene editing and nanotheranostics (**Figure** **1**). Recent developments of CRISPR/Cas technology are first summarized, providing an overview of advancements and concerns. We subsequently discuss the potential markers for detecting viral hepatitis and HCC and the emerging CRISPR‐based diagnostic (CRISPR‐Dx) platforms. Therapeutic targets and delivery vectors to apply CRISPR/Cas technology for treating both diseases are then highlighted. As the field continues perfecting CRISPR/Cas gene editing, we end the review with our perspectives on how to engineer a safe, efficient, and specific CRISPR/Cas system for liver gene editing and downstream diseases. Leveraging these aspects, this review is intended to highlight the technological advances with improving strategies to sketch potential CRISPR/Cas designs for gene editing in viral hepatitis and HCC, which may add to the armamentarium of tackling challenging liver diseases.

## 2 Current Development of CRISPR Gene Editors

### 2.1 General Mechanisms and Development of CRISPR Systems

The CRISPR/Cas system is originally part of bacterial immunity and recently developed for gene editing, which enables precise gene engineering and facilitates genomic studies in the mammalian system.

A typical CRISPR/Cas system generally contains a guide RNA (gRNA) and a corresponding Cas RNA‐guided nuclease (RGN). In bacteria, the CRISPR genes encode various short spacers and repeats. The spacers are acquired from exogenous DNA sequences captured by bacteria and act as a “blacklist” in the immune system. The short direct repeats contain palindromic sequences to form a hairpin, which can be processed into functional CRISPR RNA (crRNA) and trans‐activating crRNA (tracrRNA). The CRISPR genes are adjacent to highly conserved CRISPR‐associated (Cas) genes, organized in an operon expression system to perform spacer acquisition, crRNA processing, target cleavage, and other functions.^[^ ^8^ ^]^ Under the guidance of crRNA/tracrRNA, the Cas RGN can recognize and disrupt the exogenous sequence to defend against foreign infections.^[^ ^9^ ^]^

When adapted to the mammalian cell system, this RNA‐guided targeting and cleavage capability allow the CRISPR/Cas system to modify a specific region of the genome. To date, various engineered CRISPR/Cas systems have been validated across different cell lines and animal models to knock out or correct disease‐associated mutations, regulate gene expression, and screen functional gene signatures.^[^ ^10^ ^]^

#### 2.1.1 CRISPR/Cas‐Mediated Gene Editing: Using CRISPR/Cas9 as an Example

In general, the CRISPR/Cas gene editing recognizes the target, induces cleavage, and then triggers the DNA repair mechanism. Using the CRISPR/Cas9 system as an example, the Cas9 RGN recognizes a short protospacer adjacent motif (PAM) in the target and then performs specific base pairing with a crRNA/tracrRNA hybrid or a 20‐nt protospacer‐carrying single gRNA (sgRNA).^[^ ^11^ ^]^ The Cas9 enzyme acts as an endonuclease and cuts both complementary and non‐complementary strands, causing a double‐strand DNA break (DSB). The DNA repair mechanism is subsequently initiated via error‐prone non‐homologous end‐joining (NHEJ) or precise homology‐directed repair (HDR).^[^ ^10b^ ^]^ NHEJ‐induced repair tends to produce staggered ends, for which undesirable errors such as genetic insertions and deletions (indels) may occur. By contrast, when a homologous donor template is available, HDR can introduce specific‐site insertions, deletions, nucleotide substitutions, and genomic sequence rearrangements.^[^ ^10b^ ^]^ Therefore, HDR‐mediated CRISPR/Cas editing is often used for accurate genetic corrections. Since NHEJ is error prone, while HDR has higher fidelity, a comprehensive understanding of the underlying DNA repair mechanisms triggered after CRISPR/Cas gene editing warrants more studies to better design a proper CRISPR/Cas approach for different editing desires.

#### 2.1.2 Classification of CRISPR/Cas Systems

There are two classes of CRISPR/Cas with different compositions of interference effectors, and they have been engineered as toolkits for gene editing.^[^ ^12^ ^]^ The class 1 system has multiple subunit effector complexes, whereas the class 2 system (the focus in this review) possesses single‐protein effectors and is used for mammalian cell gene editing. Nowadays, majority of the field focuses on the class 2 systems and their derived variants, including DNA‐ and RNA‐targeting CRISPR systems. These findings adequately elucidate the vast diversity of functionality and development history of CRISPR/Cas (**Table** **1**).^[^ ^8^ ^]^

**Table 1:** Comparison of the representative CRISPR/Cas systems (Cas9, Cas12, Cas13, and Cas14)

Name	Size (amino acids)	Enzymatic domains	gRNA length (nt)	Target	PAM	Cleavage mechanism	Cutting site	Ref.
Cas9	1000−1600	HNH and RuvC	100	dsDNA, RNA	5' NGG; G‐rich	Blunt ended DSB	Proximal to recognition site	^[^ ^145^ ^]^
Cas12	1100−1300	RuvC	42−44	dsDNA	5' TTN; T‐rich	Staggered ended DSB in 5' overhangs target DNA and collateral activity	Distal from the recognition site	^[^ ^146^ ^]^
Cas13	900−1300	HEPN	52−66	RNA	3' A, U, or C	Specific RNA cleavage and collateral activity	Distal from the recognition site	^[^ ^36a^ ^]^
Cas14	400−700	RuvC	140	dsDNA and ssDNA	For dsDNA targeting: 5' TTN; T‐rich For ssDNA targeting: no limits	Super‐specific ssDNA cleavage and collateral activity	Distal from the recognition site	^[^ ^18^ ^]^

Among DNA‐targeting CRISPR/Cas systems, Cas9 with HNH and RuvC nuclease domains is one of the most intensively studied ones. To date, the Cas9 RGN from *Streptococcus pyogenes* (SpCas9) is widely used for DNA gene editing.^[^ ^13^ ^]^ Other Cas9 orthologues, such as *Staphylococcus aureus* Cas9 (SaCas9), *Streptococcus thermophilus* Cas9 (StCas9), *Neisseria meningitidis* Cas9 (NmCas9), and the SpCas9 variants, have also been optimized.^[^ ^14^ ^]^

Besides CRISPR/Cas9, other CRISPR/Cas systems with unique properties have been explored for their capability of gene editing.^[^ ^12^ ^]^ For example, Cas12, another effective DNA‐targeting subtype, can also disrupt double‐stranded DNA (dsDNA) sequences. The commonly used Cas12a (also known as Cpf1) and lately discovered Cas12b are both the members of Cas12 family.^[^ ^15^ ^]^ Unlike Cas9, Cas12 has a relatively smaller physical size with RuvC domain and is only guided by a single and short crRNA. It prefers a T‐rich PAM at the 5’ end of the protospacer and produces a sticky end distal to the PAM site.^[^ ^8^ ^]^

While as a representative RNA‐targeting system, Cas13 with a pair of higher eukaryotes and prokaryotes nucleotide‐binding (HEPN) nuclease domains was discovered in *L. shahii* bacterial pathogens.^[^ ^16^ ^]^ Cas13a (also known as C2c2) is the typical one, and Cas13b, Cas13d (CasRx), and other members were later discovered and showed a broad range of potential applications.^[^ ^8^, ^17^ ^]^

Doudna and co‐workers recently identified Cas14 (also known as Cas12f) with a smaller size of 400 to 700 amino acids.^[^ ^18^ ^]^ It can target both dsDNA and single‐stranded DNA (ssDNA) without any PAM preference. Cas14 has been used to detect single‐nucleotide polymorphisms, which is of great clinical significance for the early diagnosis of various genetic diseases and cancers.

#### 2.1.3 Development of CRISPR/Cas Systems

**Figure 2:**
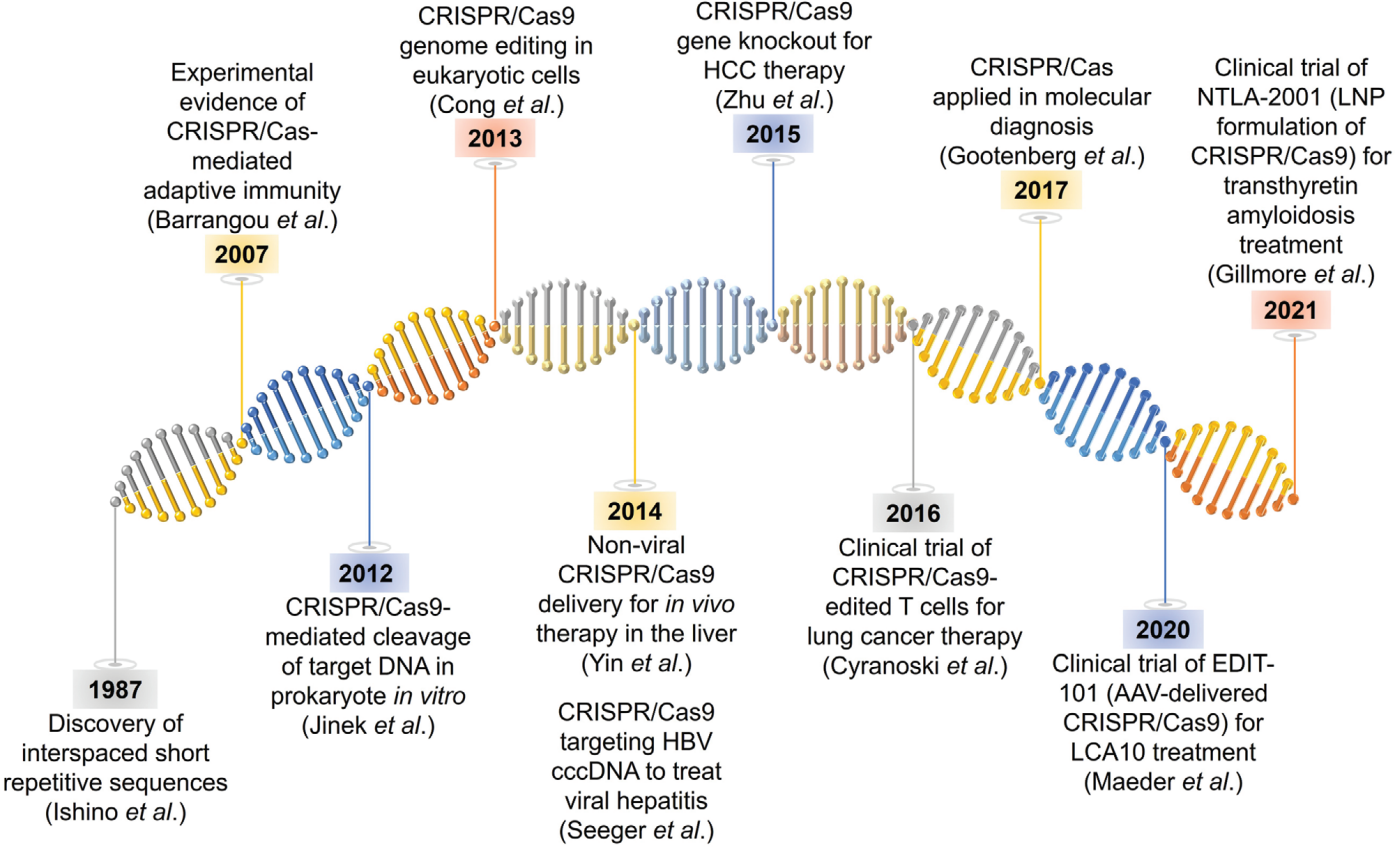
Timeline of developing breakthroughs of CRISPR/Cas systems and liver gene editing.

In the past few years, significant efforts have been made in gene editing to improve the performance of the discovered CRISPR/Cas system and explore more applications in both diagnostics and therapeutics, including liver gene editing (**Figure** **2**). In 1987, Nakata and co‐workers discovered interspaced short repetitive sequences downstream of the *Escherichia coli* iap gene.^[^ ^19^ ^]^ These interspaced repeats were later reported in other bacteria and archaea and formally termed CRISPR.^[^ ^20^ ^]^ Cas genes were also identified to be invariably adjacent to the CRISPR loci.^[^ ^20a^ ^]^ In 2007, CRISPR/Cas was experimentally confirmed as part of bacterial immune system for adaptive immunity.^[^ ^21^ ^]^ In 2012, CRISPR/Cas9 was used to cut the target DNA in prokaryotic cells, symbolizing the burst of CRISPR/Cas gene engineering.^[^ ^11b^ ^]^ One year later, CRISPR/Cas9 was applied in eukaryotic cells.^[^ ^22^ ^]^ In 2014, Yin *et al*. used hydrodynamic injection for CRISPR gene correction to treat hereditary tyrosinemia type I in the liver.^[^ ^23^ ^]^ It was one of the first reports of non‐viral delivery for CRISPR/Cas9 in vivo application in the liver. In the same year, CRISPR/Cas9 was applied to treat viral hepatitis.^[^ ^24^ ^]^ This report showed that CRISPR/Cas9 gene editing achieved eightfold infectious HBV deletion in vitro. In 2015, CRISPR/Cas9 was used for gene knockout in HCC cells, remarkably inhibiting tumor growth.^[^ ^25^ ^]^ In 2016, the CRISPR‐based clinical trial was initiated using gene‐edited T cells to treat lung cancer.^[^ ^26^ ^]^ In 2017, Zhang and co‐workers developed a Cas13‐based diagnostic platform and thus established CRISPR/Cas molecular diagnosis.^[^ ^17a^ ^]^ In 2020, in vivo CRISPR/Cas‐based therapy (EDIT‐101) was carried out to treat Leber's congenital amaurosis 10 (LCA10).^[^ ^27^ ^]^ In 2021, the clinical data of in vivo CRISPR/Cas9‐based trial was published.^[^ ^28^ ^]^ The CRISPR/Cas9‐based NTLA‐2001 therapy demonstrated its positive results to cure transthyretin amyloidosis.

CRISPR technology opens up a new avenue leading gene engineering toward clinical applications. Significant numbers of CRISPR/Cas‐based gene‐editing strategies have entered clinical trials (**Table** **2**). In 2016, the CRISPR/Cas‐based clinical trial was started in China (NCT02793856). In this *ex vivo* trial to treat non‐small cell lung cancer (NSCLC), the T cells were engineered via CRISPR‐mediated gene knockout of programmed death ligand‐1 (PD‐L1). This gene knockout blocked the PD‐L1/programmed death‐1 (PD‐1) pathway, and these engineered T cells were subsequently expanded and given back to the patient. The current clinical results demonstrated its therapeutic safety and potential for large‐scale trials.^[^ ^26^, ^29^ ^]^ On the other hand, EDIT‐101 got permission for clinical trials (NCT03872479) in 2019. This virus‐delivered CRISPR/Cas system was designed to disrupt the CEP290 gene for treating LCA10.^[^ ^30^ ^]^ The treatment of the first patient recruited for the EDIT‐101 trial was given in early 2020, remarking a significant CRISPR‐based clinical translation milestone.

**Table 2:** CRISPR‐based clinical trials (adapted from https://clinicaltrials.gov, accessed on 23 July 2021)

NCT number	Title	Condition or disease	Targets	CRISPR interventions	Additional interventions	Status	Phase
02793856	PD‐1 knockout engineered T cells for metastatic non‐small cell lung cancer	Metastatic non‐small cell lung cancer	PD‐1 in autologous T cells	*Ex vivo* CRISPR/Cas9 editing for CAR T therapy	Cyclophosphamide	Completed	Phase 1
02863913	PD‐1 knockout engineered T cells for muscle‐invasive bladder cancer	Invasive bladder cancer stage IV	PD‐1 in autologous T cells	*Ex vivo* CRISPR/Cas9 editing for CAR T therapy	Cyclophosphamide	Withdrawn	Phase 1
02867332	PD‐1 knockout engineered T Cells for metastatic renal cell carcinoma	Metastatic renal cell carcinoma	PD‐1 in autologous T cells	*Ex vivo* CRISPR/Cas9 editing for CAR T therapy	Cyclophosphamide	Withdrawn	Phase 1
02867345	PD‐1 knockout engineered T cells for castration resistant prostate cancer	Castration‐resistant prostate cancer	PD‐1 in autologous T cells	*Ex vivo* CRISPR/Cas9 editing for CAR T therapy	Cyclophosphamide	Withdrawn	
03044743	PD‐1 knockout EBV‐CTLs for advanced stage Epstein–Barr virus (EBV)‐associated malignancies stage IV gastric carcinoma	EBV positive advanced stage malignancies	PD‐1 in autologous T cells	*Ex vivo* CRISPR/Cas9 editing for CAR T therapy	Fludarabine, cyclophosphamide, interleukin‐2	Recruiting	Phase 1 Phase 2
03057912	A safety and efficacy study of TALEN and CRISPR/Cas9 in the treatment of HPV‐related cervical intraepithelial neoplasia I	HPV‐related malignant neoplasm	HPV16 and HPV18 E6/E7 DNA	Administration of CRISPR/Cas9 gel and TALEN gel consists of corresponding plasmids	TALEN	Unknown	Phase 1
03081715	PD‐1 knockout engineered T cells for advanced esophageal cancer	Esophageal cancer	PD‐L1 in T cells	*Ex vivo* CRISPR/Cas9 editing for T cell therapy		Completed	Phase 2
03164135	Safety of transplantation of CRISPR CCR5‐modified CD34^+^ cells in HIV‐infected subjects with hematological malignances	HIV‐1 infection	CCR5 in CD34^+^ hematopoietic stem/progenitor cells	*Ex vivo* CRISPR/Cas9 editing		Recruiting	Not Applicable
03166878	A study evaluating UCART019 in patients with relapsed or refractory CD19^+^ leukemia and lymphoma	B cell leukemia and lymphoma	TCR and B2M in CAR T cells	Lentiviral transduction of CAR and CRISPR RNA electroporation: UCART019		Recruiting	Phase 1 Phase 2
03332030	Stem cells in NF1 patients with tumors of the central nervous system	Neurofibromatosis type 1 (NF1)	NF1 in induced pluripotent stem cells (iPSCs)	*Ex vivo* CRISPR/Cas9 editing in iPSCs		Suspended	
03342547	Identification of host factors of norovirus infections in mini‐gut model	Gastrointestinal infection	Host essential and restrictive factors on Norovirus‐infected mini‐guts	Genome‐wide genetic screening by CRISPR knockout and gain‐of‐function CRISPR SAM		Unknown	Not Applicable
03398967	A feasibility and safety study of universal dual specificity CD19 and CD20 or CD22 CAR T cell immunotherapy for relapsed or refractory leukemia and lymphoma	B cell leukemia and lymphoma	CD19 and CD20 or CD22 in CAR T cells	*Ex vivo* CRISPR/Cas9 editing for CAR T therapy		Recruiting	Phase 1 Phase 2
03399448	NY‐ESO‐1‐redirected CRISPR (TCRendo and PD‐1)‐edited T cells (NYCE T cells)	Multiple myeloma	TCR and PD‐1 in CAR T cells	Lentiviral NY‐ESO‐1 transduction and CRISPR/Cas9 electroporation for CAR T therapy	Cyclophosphamide, fludarabine	Terminated	Phase 1
03538613	Study of people with metastatic gastrointestinal epithelial cancer administering tumor‐infiltrating lymphocytes in which the gene‐encoding CISH was inactivated using the CRISPR/Cas9 system	Metastatic gastrointestinal epithelial cancer	Cytokine‐induced SH2 protein (CISH) in lymphocyte cells	CRISPR/Cas9 editing in lymphocyte cells	Cyclophosphamide, fludarabine, aldesleukin	Withdrawn	Phase 1 Phase 2
03545815	Study of CRISPR‐Cas9 mediated PD‐1 and TCR gene‐knocked out mesothelin‐directed CAR T cells in patients with mesothelin positive multiple solid tumors	Mesothelin positive multiple solid tumors in adults	PD‐1 and TCR in CAR T cells	*Ex vivo* CRISPR/Cas9 editing for CAR T therapy		Recruiting	Phase 1
03606486	Lavage of the uterine cavity for diagnosis of ovarian cancer	High grade ovarian serous adenocarcinoma	TP53 gene	CRISPR‐Duplex sequencing	CRISPR‐duplex sequencing	Recruiting	Not Applicable
03655678	A safety and efficacy study evaluating CTX001 in subjects with transfusion‐dependent *β*‐thalassemia	Transfusion‐dependent *β*‐thalassemia (TDT)	BCL11A in autologous CD34^+^ human hematopoietic stem and progenitor cells (hHSPCs)	*Ex vivo* CRISPR/Cas9 editing to modify autologous CD34^+^ hHSPCs: CTX001		Recruiting	Phase 1 Phase 2
03690011	Cell therapy for high‐risk T‐Cell malignancies using CD7‐specific CAR expressed on autologous T cell	High‐risk T‐cell malignancies	CD7 gene in autologous T cells	*Ex vivo* CRISPR/Cas9 editing for CAR T therapy	Fludarabine, Cytoxan	Not yet recruiting	Phase 1
03728322	iHSCs with the gene correction of HBB intervent subjects with *β*‐thalassemia mutations	Thalassemia	HBB in patient‐specific induced hepatic stem cells (iHSCs)	*Ex vivo* CRISPR/Cas9 editing for gene correction in patient‐specific iHSCs		Unknown	Early Phase 1
03745287	A safety and efficacy study evaluating CTX001 in subjects with severe sickle cell disease	Sickle cell disease, hematological diseases, hemoglobinopathies	BCL11A gene in autologous CD34^+^ hHSPCs	*Ex vivo* CRISPR/Cas9 editing and CAR T therapy: CTX001		Recruiting	Phase 1 Phase 2
03747965	Study of PD‐1 gene‐knocked out mesothelin‐directed CAR T cells with the conditioning of PC in mesothelin positive multiple solid tumors	Mesothelin positive multiple solid tumors in adults	PD‐1 in CAR T cells	*Ex vivo* CRISPR/Cas9 editing for CAR T therapy	Paclitaxel, cyclophosphamide	Unknown	Phase 1
03855631	Exploiting epigene editing in kabuki syndrome: a new route toward gene therapy for rare genetic disease	Kabuki syndrome 1	KMT2D gene in primary cells isolated from affected patients	*Ex vivo* CRISPR/Cas9 editing		Active, not recruiting	
03872479	Single ascending dose study in participants with LCA10	Leber congenital amaurosis (LCA) 10	Centrosomal protein 290 (CEP290)	Single escalating doses of CRISPR/Cas9 targeting CEP290 (EDIT‐101) via subretinal injection		Recruiting	Phase 1 Phase 2
04035434	A safety and efficacy study evaluating CTX110 in subjects with relapsed or refractory B‐cell malignancies (CARBON)	Relapsed or refractory B‐cell malignancies	CD19 in allogeneic T cells	*Ex vivo* CRISPR/Cas9 editing in allogeneic T cells: CTX110		Recruiting	Phase 1
04037566	CRISPR (HPK1)‐edited CD19‐specific CAR T cells (XYF19 CAR T cells) for CD19^+^ leukemia or lymphoma	Relapsed or refractory malignancies	HPK1 in CAR T cells	Lentiviral CD19 transduction and CRISPR/Cas9 electroporation for CAR T therapy	Cyclophosphamide, fludarabine	Recruiting	Phase 1
04074369	Evaluation of CRISPR‐based test for the rapid Identification of TB in pulmonary tuberculosis suspects	Pulmonary tuberculosis	Mycobacterium tuberculosis (MTB) in sputum or bronchoalveolar lavage fluid (BALF) samples	CRISPR/Cas detection		Recruiting	
04178382	Effect of PCR‐CRISPR/Cas12a on the early anti‐infective schemes in patients with open air pneumonia	Open air pneumonia patients with severe sepsis	Microorganisms in alveolar lavage fluid samples guided by PCR analysis	Combined detection of PCR and CRISPR/Cas12a in the alveolar lavage fluid		Recruiting	Not Applicable
04208529	A long‐term follow‐up study in subjects who received CTX001	Subjects who received CTX001 in Study CTX001‐111 (NCT03655678) or Study CTX001‐121 (NCT03745287).	BCL11A gene in autologous CD34^+^ hHSPCs	CTX001		Enrolling by invitation	
04244656	A safety and efficacy study evaluating CTX120 in subjects with relapsed or refractory multiple myeloma	Relapsed or refractory multiple myeloma	B‐cell maturation antigen (BCMA)‐encoding gene in allogeneic T cells	*Ex vivo* CRISPR/Cas9 editing for CAR T therapy: CTX120		Recruiting	Phase 1
04417764	TACE combined with PD‐1 knockout engineered T cell in advanced hepatocellular carcinoma	Advanced hepatocellular carcinoma	PD‐1 in autologous T cells	*Ex vivo* CRISPR/Cas9 editing for CAR T therapy	Transcatheter arterial chemoembolization (TACE)	Recruiting	Phase 1
04426669	A study of metastatic gastrointestinal cancers treated with tumor infiltrating lymphocytes in which the gene encoding the intracellular immune checkpoint CISH is inhibited using CRISPR genetic engineering	Gastrointestinal cancers	Intracellular immune checkpoint CISH in tumor infiltrating lymphocytes (TILs)	*Ex vivo* CRISPR/Cas9 editing	Cyclophosphamide, fludarabine and Aldesleukin	Recruiting	Phase 1 Phase 2
04438083	A safety and efficacy study evaluating CTX130 in subjects with relapsed or refractory renal cell carcinoma	Renal cell carcinoma	CD70 in allogeneic T cells	*Ex vivo* CRISPR/Cas9 editing for CAR T therapy: CTX130		Recruiting	Phase 1
04502446	A safety and efficacy study evaluating CTX130 in subjects with relapsed or refractory T or B cell malignancies	T cell lymphoma	CD70 in allogeneic T cells	CTX130		Recruiting	Phase 1
04535505	Pathogenic bordetella rapid detection	Pertussis	Drug resistant genes in pathogenic bodella	CRISPR/Cas detection for single point mutations	Detection pathogenic pertussis by cross primer constant temperature amplification (CPA)	Not yet recruiting	
04535648	Detection of enterovirus genotypes by CRISPR technology	Enterovirus infections	Genotypes of enterovirus in samples of feces, blood and cerebrospinal fluid	Non‐invasive CRISPR detection		Not yet recruiting	
04557436	TT52CAR19 therapy for B‐cell acute lymphoblastic leukemia (B‐ALL) (PBLTT52CAR19)	B acute lymphoblastic leukemia	CD52 and TRAC in allogenic engineered human T cells	Lentiviral transduction into anti‐CD19 chimeric antigen receptor (CAR19) and CRISPR/Cas9 electroporation for CAR T therapy: PBLTT52CAR19		Recruiting	Phase 1
04560790	Safety and efficacy of CRISPR/Cas9 mRNA instantaneous gene‐editing therapy to treat refractory viral keratitis	Viral keratitis	Herpes simplex virus type I (HSV‐1)	Single escalating doses of BD111 CRISPR/Cas9 mRNA via corneal injection		Active, not recruiting	Phase 1 Phase 2
04601051	Study to evaluate safety, tolerability, pharmacokinetics, and pharmacodynamics of NTLA‐2001 in patients with hereditary transthyretin amyloidosis with polyneuropathy (ATTRv‐PN)	Hereditary transthyretin amyloidosis	Transthyretin (TTR) gene in the liver	LNP‐delivered CRISPR/Cas9 editing via intravenous administration: NTLA‐2001		Recruiting	Phase 1
04637763	CRISPR‐edited allogeneic anti‐CD19 CAR T cell therapy for relapsed/refractory B cell non‐Hodgkin lymphoma	Relapsed/refractory B cell non‐Hodgkin lymphoma	CD19 in autologous T cells	*Ex vivo* CRISPR/Cas9 editing for CAR T therapy: CB‐010	Cyclophosphamide, fludarabine	Recruiting	Phase 1
04774536	Transplantation of clustered regularly interspaced short palindromic repeats modified hematopoietic progenitor stem cells (CRISPR_SCD001) in patients with severe sickle cell disease	Sickle cell disease	Mutant HBB gene in autologous CD34+ cells	IV administration of CRISPR_SCD001 following myeloablative conditioning with busulfan		Not yet recruiting	Phase 1 Phase 2
04819841	Gene correction in autologous CD34+ hematopoietic stem cells (HbS to HbA) to treat severe sickle cell disease (CEDAR)	Sickle cell disease	Mutant HBB gene in hHSPCs	IV administration of GPH101 following myeloablative conditioning with busulfan		Not yet recruiting	Phase 1 Phase 2
04925206	A multicenter, open label phase 1 study to evaluate the safety and efficacy of a single dose of autologous CRISPR‐Cas9‐modified CD34+ human hematopoietic stem and progenitor cells (hHSPCs) in subjects with transfusion dependent *β*‐thalassaemia	Transfusion dependent beta‐thalassaemia	BCL11A gene in autologous CD34+ cells	IV administration of ET‐01 following myeloablative conditioning with busulfan		Not yet recruiting	Phase 1

For liver diseases, CRISPR‐based nanotheranostics is still at an early stage. Nevertheless, the momentum of CRISPR/Cas studies has pushed forward theranostics of liver diseases, including viral hepatitis and HCC to get closer to clinical applications. In the following sections, we will discuss how CRISPR/Cas technologies could be integrated with nanotheranostics to design innovative CRISPR‐based strategies for detecting and treating viral hepatitis and HCC.

### 2.2 DNA‐Targeting CRISPR System Used in Liver Disease Theranostics

**Figure 3:**
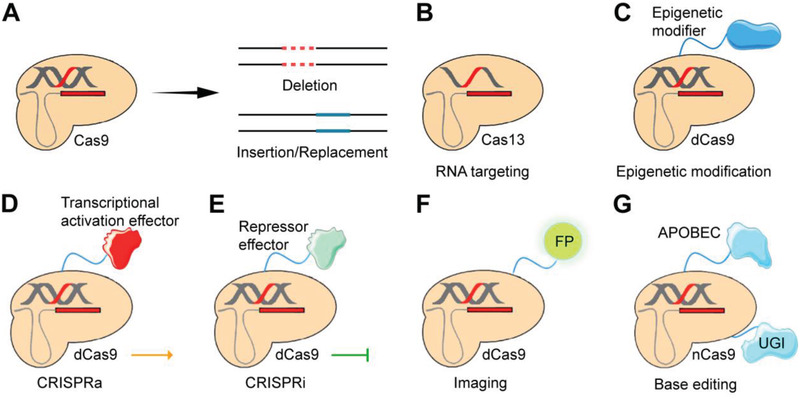
Examples of CRISPR/Cas‐mediated gene editing technology. A) Primarily, Cas9 performs gene editing to achieve gene deletion and insertion/replacement. B) Some CRISPR systems like Cas13 orthologs can target RNA instead of DNA. C) dCas9 can be engineered with epigenetic modifiers to induce epigenomic editing. D,E) dCas9 engineered with trans‐effectors can establish CRISPRa connected with the activation domain or CRISPRi connected with the repressor domain. F) CRISPR imaging is achieved by fusing a fluorescence protein (FP). G) CRISPR/Cas9 base editing is based on nCas9 integrated with UGI and APOBEC1.

CRISPR/Cas‐based DNA targeting has been widely studied since when the initial CRISPR/Cas gene editor, SpCas9, was reported. As one of the most common DNA‐targeting gene editors, CRISPR/Cas9 systems have been used for gene deletion and insertion/replacement in multiple fields (**Figure** **3**A).^[^ ^13^, ^31^ ^]^

Especially, DNA‐targeting CRISPR systems have been an active and promising field of liver‐associated research.^[^ ^32^ ^]^ As a representative example, a CRISPR/Cas9‐mediated metabolic pathway reprogramming achieved in vivo gene editing to delete metabolic enzyme hydroxyphenylpyruvate dioxygenase.^[^ ^33^ ^]^ CRISPR‐edited hepatocytes displayed a reversion from hereditary tyrosinemia to benign tyrosinemia, making treated mice asymptomatic within 8 weeks. In the following section, more and more mentioned studies supported the potential of CRISPR‐based approaches in liver studies.

### 2.3 RNA‐Targeting CRISPR Gene Editors

RNA can also exploit genetic information and participate in various cellular activities.^[^ ^16b^ ^]^ Transcriptional homeostasis is essential for maintaining physiological functions, so RNA targeting to correct abnormal RNA levels is one option for treating diseases. CRISPR‐mediated RNA editing is a potential strategy. Distinct from permanent alterations caused by DNA editing, RNA editing is reversible and thus suitable for temporary modifications. The major advantage of RNA editing is to minimize genetic and mechanistic complications,^[^ ^13^, ^16^, ^31^, ^34^ ^]^ especially appealing for its potential in treating RNA virus infections. Notably, as HCV is an RNA virus, the expansive RNA‐targeting CRISPR gene editors have great potentials for theranostics of HCV‐induced viral hepatitis. CRISPR/Cas‐mediated RNA editing can perform posttranscriptional silencing for HCC therapy.

Among CRISPR/Cas systems, the type VI system can be RNA targeting. The Cas13 family is one of the most representative systems to expand RNA‐targeting applications (Figure 3B).^[^ ^17b^ ^]^ Cas13a and Cas13b have been used for RNA editing, RNA imaging, splicing modulation, nucleic acid detection, and so on.^[^ ^8^, ^17^, ^35^ ^]^ Remarkably, Zhang and co‐workers reported a deactivated CRISPR/Cas13‐programmable adenosine (A)‐to‐inosine (I) replacement (REPAIR) system and a later cytidine (C)‐to‐uridine (U) exchange (RESCUE) system.^[^ ^36^ ^]^ These two systems had no restrictive targeting sequences and precisely corrected pathogenic mutations of transcripts. Furthermore, Li *et al*. fused dCas13b and N6‐methyladenosine (m^6^A) demethylase AlkB homolog 5 (ALKBH5) as the designed dm^6^ACRISPR system.^[^ ^37^ ^]^ The dm^6^ACRISPR system targeted and then demethylated mRNA, inducing efficient demethylation of m^6^A oncogene transcripts to inhibit tumor proliferation. As for the liver applications, a study innovatively used Cas13d in the mouse liver for PTEN and PCSK9 gene knockdown to modulate metabolic functions.^[^ ^38^ ^]^ Results showed more than 15% knockdown, illustrating Cas13‐based RNA‐targeting strategy as a powerful method to inactivate genes in vivo.

Besides, Cas9 can act as an RNA‐guided ribonuclease too. A PAM‐presenting oligonucleotide (PAMmer) designed for specific RNA sequences can guide Cas9 to target RNA sites. This RNA‐targeting Cas9 system (termed RCas9) has been used to visualize and eliminate targeted RNAs in vitro.^[^ ^39^ ^]^ Lei and co‐workers developed a CRISPR‐mediated fluorescence in situ hybridization in live cells (CRISPR LiveFISH).^[^ ^40^ ^]^ They integrated dCas9‐EGFP fusion protein and Cy3‐labeled gRNA to form fluorescent ribonucleoprotein (fRNP). CRISPR LiveFISH is verified to be used for RNA editing and localization. Notably, a Cas9 orthologue termed *Francisella novicida* Cas9 (FnCas9) was also reported with ribonuclease activity. This system was previously applied to target HCV in eukaryotic cells and achieved over 50% virus inhibition.^[^ ^41^ ^]^

### 2.4 Deactivated CRISPR Systems for Nucleic Acid Manipulation

By mutating the two nuclease domains, HNH and RuvC, the catalytically deactivated Cas9 (dCas9) can target the locus without cleaving the site. Creating a single mutation on one of the nuclease domains can make Cas9 nickase (nCas9) only cut one targeted strand. Similarly, the generations of dead Cas12 (dCas12) and dead Cas13 (dCas13) can also be achieved through mutation of the RuvC and the HEPN nuclease domains, respectively. These deactivated CRISPR systems directly manipulate the transcription or the recruitment of effector proteins to regulate the gene expression (Figure 3C).^[^ ^42^ ^]^ Furthermore, this way of genetic manipulation has been under exploration in the field of the liver.

Integration of a deactivated CRISPR/Cas with different effectors can further generate a targeted gene modifier. Many CRISPR activators (CRISPRa) and CRISPR repressors (CRISPRi) have been reported for gene regulation applications (Figure 3D,E).^[^ ^1c^ ^]^ For instance, Wang *et al*. synthesized dCas9 epi‐suppressors by fusing dCas9 to three epigenetic suppressor genes for HCC therapy.^[^ ^43^ ^]^ They used this system to target the granulin (GRN), a promotive factor of pluripotent mitogen and growth in tumor progression. In Hep3B hepatoma cells, the epigenetic modification via tethering catalytically inactive Cas9 with DNMT3a, EZH2, and KRAB domains was verified to inhibit tumor growth efficiently.

Besides, the dCRISPR family can be integrated with reporters like a fluorescent protein (FP) for imaging (Figure 3F).^[^ ^44^ ^]^ This imaging technology offers extraordinary potential in dynamic visualization within living cells or even in vivo. More recently, dCRISPR systems have been under unprecedented revolution with the development of CRISPR/Cas base editors (BEs), which are generated by nCas9 fused with uracil glycosylase inhibitor (UGI) and cytosine deaminase, APOBEC1 (Figure 3G).^[^ ^45^ ^]^ BE‐mediated gene manipulation is promising for correcting point‐mutated genetic disorders, and more BEs have been reported with the adaptation of fusing with adenosine or cytidine deaminases, performing four transition mutations: C‐G to T‐A or A‐T to G‐C substitutions. Recently, Yang *et al*. used the SpCas9‐based BEs to eliminate HBV in the liver and successfully edited base in an in vitro HBV infection model.^[^ ^46^ ^]^ They introduced point mutations to integrated HBV DNAs and covalently closed circular DNAs (cccDNAs), indicating the potential to treat HBV via CRISPR BE systems. This study has demonstrated the potential use of base editing technology in the liver.

Collectively, these deactivated CRISPR systems for diverse gene manipulations demonstrated their potential to be used for viral hepatitis and HCC in diagnostic imaging and epigenetic therapy. More systematic investigations have been designed to balance editing efficiency and side effects in applications of viral hepatitis and HCC.

### 2.5 Concerns and Obstacles

Although the CRISPR/Cas technology is promising in treating genetic‐associated mutations and diseases,^[^ ^47^ ^]^ some significant challenges and obstacles hinder the translation of this technology, especially on its off‐targeting and safety.^[^ ^48^ ^]^

#### 2.5.1 Off‐Targeting Effects

Theoretically, the CRISPR/Cas system only disrupts the targeted DNA that was recognized by its gRNA.^[^ ^49^ ^]^ However, in some cases, other sites that are not fully complementary to the protospacer may also be cleaved by Cas RGNs, known as the off‐targeting effects.^[^ ^21^, ^50^ ^]^ Undesired editing at the off‐target sites severely impacts the editing efficacy and may harm cell survival and other physical activities.

Many efforts have been attributed to reducing off‐target effects, including optimizing the gRNA designs, using a pair of nCas9. The gRNAs can be modified with sequence truncation, chemical modifications.^[^ ^51^ ^]^ In addition, the protospacer sequence also impacts the editing specificity. Many prediction tools have been reported to guide the design of CRISPR/Cas system. For example, “CRISPOR” website (http://crispor.org) is a tool to help gRNA design according to a scoring algorithm evaluating potential off‐target and on‐target activities in over 150 genomes.^[^ ^52^ ^]^ Recently, DeepSpCas9 (http://deepcrispr.info/DeepSpCas9), a deep learning‐based model, was developed to predict SpCas9 activities with various targeting gRNAs.^[^ ^53^ ^]^ Besides, bioinformatics techniques and advanced sequencing techniques are on the way to guide the design of CRISPR/Cas toolkit to reduce potential off‐target effects.^[^ ^51^, ^54^ ^]^

Proper engineering of Cas proteins is another solution. Slaymaker *et al*. generated “enhanced specificity” SpCas9 (eSpCas9) variants from the neutralization of positively charged residues within the nontarget strand groove of SpCas9.^[^ ^55^ ^]^ These variants showed weaker binding between the nontarget strand and the target one, achieving higher fidelity but maintaining robust on‐target cleavage. Hu *et al*. generated another Cas9 variant with expanded PAM compatibility (xCas9) through phage‐assisted continuous evolution.^[^ ^11a^ ^]^ They identified that xCas9 broadened the PAM scope to NGG, NG, GAA, and GAT for more precise targeting and showed genome‐wide off‐target activity than SpCas9.

Choosing DSB‐induced repairing pathways is another improving direction. HDR‐induced gene editing with high fidelity Cas enzyme can improve targeted integrations, while NHEJ is prone to induce various indels and thus increase the off‐target risk.^[^ ^22^, ^56^ ^]^ Recently, a third repair mechanism named microhomology‐mediated end‐joining (MMEJ) was reported and enable efficient targeted integration of large DNA fragments.^[^ ^57^ ^]^ It came with high targeted editing efficiency and low off‐target effects in transfected hepatocytes, promoting a practical approach for gene manipulation in the liver.

#### 2.5.2 Immunogenicity

Potential immune reactions might engender another big concern. The introduced CRISPR/Cas systems may be identified as foreigners to generate immune responses and even activate anti‐Cas antibodies and Cas‐specific cellular responses, which are likely to induce cell death or other consequences.^[^ ^15^, ^34^, ^58^ ^]^ The immune system may cause CRISPR/Cas editing failure via graft‐versus‐host rejection and severe immune responses. Porteus’ group found that >70% of adults have antibodies of the widely‐used SaCas9 and SpCas9, warning for CRISPR‐based clinical trials with potential inflammation and even death.^[^ ^59^ ^]^ Later, another study indicated pre‐existing immunity to SpCas9 appeared in 85% of healthy volunteers.^[^ ^60^ ^]^ All these results emphasize immunogenicity as a tricky problem in CRISPR/Cas editing.

Encouragingly, these reports of warning alerted scientists to carefully design a CRISPR/Cas system for the particular application for the future clinic. CRISPR applications into clinical transformation require a thorough study of harmful immune hazards to prevent reverse immune responses and maintain high editing efficiency. For theranostics of viral hepatitis and HCC, a safe, efficient and specific CRISPR/Cas tool is necessary.^[^ ^32^, ^58^ ^]^

Although off‐target effect and immunogenicity could compromise the gene‐editing efficacy, these may be resolved with the help of other engineering approaches. Nevertheless, CRISPR/Cas gene editing holds great potential for viral hepatitis and HCC. Indeed, the promise of CRISPR/Cas as a tool for cleavage, elimination, or inactivation of hepatitis viruses and HCC‐related genes has prompted a significant number of researchers to explore the possibility to treat viral infections and HCC.

## 3 CRISPR Technology for Diagnostics of Viral hepatitis and Hepatocellular Carcinoma

As CRISPR/Cas technology enables precise gene targeting and editing, it can detect pathogenic nucleic acids. To date, many sensitive CRISPR diagnostic systems have been designed, and those could also be potentially used for the early detection of viral hepatitis and HCC. This section will introduce the current progress of technology development, highlight the advances of CRISPR diagnostic methods, and discuss their potential use in viral hepatitis and HCC diagnosis.

### 3.1 Potential Markers for Liver Disease Diagnostics

#### 3.1.1 CRISPR Screening for Liver Diseases

For liver disease diagnostics, screening and validation of pathogenic gene signatures involved in liver‐associated diseases are the first points. These have been carried out with transposon mutagenesis and RNA interference‐mediated screens,^[^ ^61^ ^]^ but the two methods are low efficient or less specific. In contrast, the CRISPR/Cas technology offers a better alternative approach with ideal cost efficiency, time consumption, and high genetic identifying capacity.^[^ ^62^ ^]^

There have been several efforts taken for CRISPR screening of viral hepatitis. Ren *et al*. reported a real‐time live‐cell reporter system, namely NIrD, with the combination of CRISPR/Cas9 gRNA library to study HCV infections. Several gene signatures, such as CLDN1, OCLN, and CD81, were involved in HCV transmission using high‐throughput sequencing analysis. This demonstrates that CRISPR/Cas9 is a powerful tool to study the response of host cells under the virus infection.^[^ ^63^ ^]^

On the other hand, both oncogenes and tumor suppressor genes (TSGs) are promising HCC diagnostic markers. Using PiggyBac (PB) transposon to deliver a gRNA library, in vivo genome‐scale screening in mice was achieved, successfully identifying several TSGs involved in liver tumorigenesis.^[^ ^64^ ^]^ Taken together, liver‐associated CRISPR screening is a fruitful direction to identify potential markers for the detections of viral hepatitis and HCC. These discovered markers have great potentials for precise diagnostics.

#### 3.1.2 Potential Markers for Viral Hepatitis Detection

To date, five certain types of viral hepatitis have been identified (**Table** **3**). All of them have different features with potential diagnostic markers. HBV and HCV are the primary focus of this review. In 1965, Blumberg *et al*. discovered hepatitis B surface antigen (HBsAg) associated with HBV in Australian aboriginal serum.^[^ ^65^ ^]^ From then on, HBsAg in the serum has become a key index for HBV active infection. This study also initiated the explorations of specific markers related to viral hepatitis. As a partly dsDNA virus, HBV has discovered a series of serological markers: HBsAg and anti‐HBs, hepatitis B e antigen (HBeAg) and anti‐HBe, and anti‐hepatitis B core antigen (HBc) IgM and IgG.^[^ ^66^ ^]^ Lately, HBV detection has extended to other markers, including the samples from serum DNA, resistance cccDNA, and so on.^[^ ^67^ ^]^ For HCV detection, HCV RNA, HCV antibody (anti‐HCV), and core antibody (anti‐HCc) have been widely used in clinics.^[^ ^68^ ^]^

**Table 3:** Five typical hepatitis viruses

Type	Classification	Viral genome	Route of transmission	Chronic infection
Hepatitis A virus (HAV)	Picornavirus	RNA	Fecal‐oral	No
Hepatitis B virus (HBV)	Hepadnavirus	DNA	Parenteral	Yes
Hepatitis C virus (HCV)	Flavivirus	RNA	Parenteral	Yes
Hepatitis D virus (HDV)	Deltavirus	RNA	Parenteral	Yes
Hepatitis E virus (HEV)	Hepevirus	RNA	Fecal‐oral	No

On‐time detection and corresponding therapy of viral hepatitis are expected to have a simple process, take up only minimal time and cost. The emerging CRISPR/Cas system can provide an opportunity as a promising precise detection tool.^[^ ^69^ ^]^ Especially, HBV DNA and HCV RNA, which are the most important indexes of virus infection, could be suitable for CRISPR/Cas diagnostics with DNA or RNA targeting. In addition, designs of high‐specific targeted sequences are necessary. Further efforts should be taken to design proper targets with high‐level affinity and binding between the disease markers and CRISPR/Cas detectors.

#### 3.1.3 Potential Markers for Hepatocellular Carcinoma Detection

The landscapes and genetic alterations in the different stages of HCC have been much clearer with published studies in the past decades,^[^ ^70^ ^]^ for which considerable factors can be considered as potential markers. Serum *α*‐fetoprotein (AFP) has been studied mainly as a reliable HCC marker in diagnostics and prognosis prediction.^[^ ^71^ ^]^ Osteopontin (OPN) was identified as a matricellular protein in the bone matrix, relevant to HCC metastasis.^[^ ^72^ ^]^ Recently, methylated DNA markers (MDMs) have been discovered as depicters for HCC‐specific genetic and epigenetic aberrations,^[^ ^73^ ^]^ so they may be suitably applied for CRISPR/Cas‐based diagnosis, surveillance, and prognosis analysis. For example, Xu *et al*. demonstrated that ctDNA methylation markers in the plasma were closely correlated to HCC DNA, providing a concept for a potential effective blood‐based diagnostic method.^[^ ^73^ ^]^ Other markers, such as CTNNB1 and TP53, have been discovered and may be suitable for CRISPR‐mediated specific HCC detection.^[^ ^74^ ^]^

In summary, a list of specific HCC markers has been studied to link with the tumor occurrence and development, and those could be potential targets for designing a CRISPR‐based HCC diagnosis (**Table** **4**). These potential markers should be selected according to HCC specificity. In addition, as the advances in CRISPR/Cas technology have driven disease diagnostics,^[^ ^32a^ ^]^ previous applications can be transferred to HCC diagnosis with mutation detection. The CRISPR/Cas detectors should be rationally designed with super sensitivity, and the reporting signals should be transformed into quantitative data.

**Table 4:** Potential detective markers of HCC

HCC molecules	Function or significance	Related pathway	Changes during HCC	Ref.
Methyltransferase‐like 3 (METTL3)	A major RNA N6‐adenosine methyltransferase (m6A), an inhibitor of cytokine signaling 2 (SOCS2) expression	m6A‐YTHDF2 (reader protein)‐dependent pathway	Upregulation	^[^ ^70^, ^147^ ^]^
Nuclear receptor coactivator 5 (NCOA5)	Positively regulating ERɑ‐mediated transcription	EMT process	Downregulation	^[^ ^70^, ^148^ ^]^
High mobility group A2 (HMGA2)	Negatively regulating Ras‐dependent activation	MAPK‐RAS pathway	Downregulation	^[^ ^149^ ^]^
Mammalian target of rapamycin (mTORC2)	Promoting fatty acid and lipid synthesis then steatosis and tumor development	mTOR pathway	Upregulation	^[^ ^150^ ^]^
Src homolog and collagen homolog 3 (Shc3)	Inducing epithelial‐mesenchymal transition (EMT) and proliferation as well as metastasis of HCC	MVP/MEK/ERK	Upregulation	^[^ ^151^ ^]^
Na^+^/Ca^2+^ exchanger 1 (NCX1)	Regulating the effect of TGF*β* on tumor migration, invasion, and metastasis via interacting with canonical transient receptor potential channel 6 (TRPC6)	TGF*β* pathway	Upregulation	^[^ ^152^ ^]^
Phospholysine inorganic pyrophosphate phosphatase (LHPP)	A protein histidine phosphatase as TSG	mTOR pathway	Downregulation	^[^ ^153^ ^]^
Long intergenic non‐coding RNA located on 1q21.2 sequence (LINC01138)	Physically interacting with insulin‐like growth factor‐2 mRNA‐binding proteins 1/3 (IGF2BP1/IGF2BP3) and arginine methyltransferase 5 (PRMT5)	Downstream PRMT5 ubiquitination and degradation	Upregulation	^[^ ^154^ ^]^
Src homology region 2 (SH2) domain‐containing phosphatase 1 (SHP‐1 or PTPN6)	Inhibiting proliferation, migration, invasion, and tumorigenicity of HCC	STAT3, NF‐*κ*B, and AKT pathway	Downregulation	^[^ ^155^ ^]^
Sterol O‐acyltransferase 1 (SOAT1)	Promoting distribution of cellular cholesterol, proliferation, and migration of HCC	TGF*β* pathway	Upregulation	^[^ ^156^ ^]^
Hepatic leukemia factor (HLF)	An oncofetal protein reactivated in HCC by SOX2 and OCT4	HLF/c‐Jun axis	Upregulation	^[^ ^157^ ^]^
Wingless‐type MMTV integration site family member 3a (Wnt3a)	A key component of the mesoderm gene in embryonic development	Wnt/*β*‐catenin pathway	Upregulation	^[^ ^158^ ^]^
Taurine upregulated gene 1 (TUG1)	Positive correlated to AFP mRNA levels in non‐hepatitis B/non‐hepatitis C HCC (NBNC‐HCC)	Undiscussed	Upregulation	^[^ ^71^ ^]^

### 3.2 CRISPR‐Based Diagnostic Platforms

The CRISPR‐Dx has been utilized to detect abnormal genetic changes for pathogen genotyping, disease monitoring, and so on.^[^ ^17^, ^35^ ^]^ Cas proteins like Cas12, Cas13, and Cas14 bind to gRNAs in a working manner as cutting both the target and the nearby sequences. When these Cas proteins recognize and cut the specific target, they turn to nonspecific nucleases that shred all the surrounding ssDNA or RNA substrates. Such collateral effects can be used to develop the CRISPR‐Dx platforms. The emerging CRISPR‐Dx systems have been studied for disease diagnostics with CRISPR‐based actors and signal reporters. As a promising candidate for the next‐generation diagnostic tool, CRISPR‐Dx can distinguish genetic alterations for early tumorigenesis, long‐lasting incubation time, and other signatures, which is essential for disease prevention or progression monitoring of viral hepatitis and HCC.

#### 3.2.1 DETECTR

**Figure 4:**
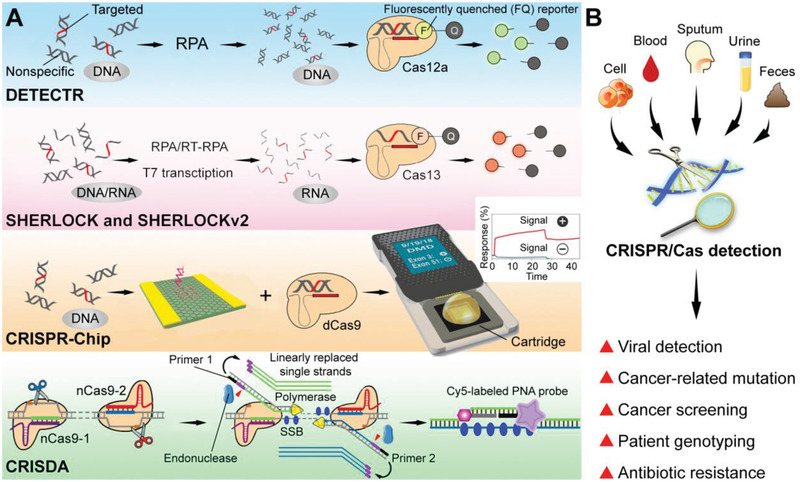
Advance of CRISPR/Cas detection systems. A) Schematic graph of representative CRISPR detectors: DETECTR,^[^ ^75^ ^]^ SHERLOCK and SHERLOCKv2,^[^ ^17^, ^35^ ^]^ CRISPR‐Chip,^[^ ^77^ ^]^ and CRISDA.^[^ ^78^ ^]^ Adapted with permission.^[^ ^75^ ^]^ Copyright 2018, Science. Adapted with permission.^[^ ^17a^ ^]^ Copyright 2017, Science. Adapted with permission.^[^ ^77^ ^]^ Copyright 2019, Springer Nature. Adapted with permission.^[^ ^78^ ^]^ Copyright 2018, Springer Nature. B) Potential applications of CRISPR detectors as diagnostic platforms.

Doudna and co‐workers developed the DNA Endonuclease Targeted CRISPR Trans Reporter (DETECTR) to integrate Cas12a, recombinase polymerase amplification (RPA), and ssDNA fluorescence reporters (**Figure** **4**A).^[^ ^75^ ^]^ The detection method relies on Cas12a's collateral effect to realize sensitive DNA detection. DETECTR was verified to differentiate subtypes of viruses (*e.g*., HPV, human papillomavirus) in both virus‐infected cell lines and clinical patient samples. These results suggested that the DETECTR platform for virus identification may be suitable for hepatitis virus detection.

#### 3.2.2 SHERLOCK and SHERLOCKv2

Zhang and co‐workers reported a Cas13‐based sensitive detector, namely Specific High‐Sensitivity Enzymatic Reporter UnLOCKing (SHERLOCK).^[^ ^17a^ ^]^ The target sequences were first amplified by isothermal amplification, including RPA and reverse transcriptase‐RPA (RT‐RPA), and the detection executed via Cas13a's trans‐cleavage on given RNA beacons. SHERLOCK was verified to specifically distinguish viruses and bacteria, diagnose human DNA genotypes and relative cancer mutations. This CRISPR‐Dx provided rapid DNA or RNA detection with attomolar sensitivity and single‐nucleotide specificity. The group recently optimized the SHERLOCK system to be a multiplexed, portable, quick, and quantitative platform (termed SHERLOCKv2).^[^ ^35^ ^]^ They incorporated Cas13, Cas12a, and Csm6 (an auxiliary CRISPR‐associated enzyme) to achieve 4‐channel simultaneous detection of several virus infections. The upgraded SHERLOCKv2 also exhibited higher sensitive and field‐deployable detection with lateral‐flow readout instead of a fluorescence reader. Persistently, the team described a protocol, Heating Unextracted Diagnostic Samples to Obliterate Nucleases (HUDSON), to pair with SHERLOCK and SHERLOCKv2 for instrument free but rapid detection.^[^ ^76^ ^]^ Potentially, SHERLOCK can be further developed to detect hepatitis viruses and identify HCC genetic markers.

#### 3.2.3 CRISPR‐Chip

CRISPR/dCas9 has also been used to develop a point‐of‐care diagnostic platform by employing dCas9's sequence‐specific binding capability. A handheld CRISPR/dCas9‐based diagnostic chip was reported (CRISPR‐Chip).^[^ ^77^ ^]^ CRISPR‐Chip, integrated dCas9 with a supersensitive graphene‐based field‐effect transistor (gFET), exploited dCas9‐mediated targeting to control gFET's changes in conductivity and electrical characteristics, thereby transforming target recognition into electrical signal outputs. The CRISPR‐Chip system could detect specific mutations in the genomic DNA samples from the patients with Duchenne muscular dystrophy without any nucleic acid amplification, and it presented rapid gene detection within 15 min. Inspired by CRISPR‐Chip, the future detection of viral hepatitis and HCC is expected to be precise and quantitative.

#### 3.2.4 CRISDA

Zhou and co‐workers reported a CRISPR/Cas9‐triggered nicking endonuclease‐mediated Strand Displacement Amplification method (namely CRISDA).^[^ ^78^ ^]^ They exploited unique conformational rearrangements of CRISPR effectors after Cas9 binding to target sequences and combined an endpoint measurement by invading peptide nucleic acid (PNA). First, they combined nCas9 (SpCas9 carrying H840A mutation) with a pair of nCas9/sgRNAs to induce nicks in both nontarget strands of the target DNA. Then, they introduced a pair of primers to induce strand displacement amplification (SDA) with the addition of polymerase, endonuclease, single‐stranded DNA binding protein (SSB), and linearly replaced single strands. Finally, they quantitatively analyzed amplification products by a PNA invasion‐mediated endpoint measurement via magnetic field (MF) and fluorescence. CRISDA exhibited attomolar sensitivity and single‐base specificity to detect breast cancer genotypes and further demonstrated sub‐attomolar sensitivity with Cas9‐mediated target enrichment. Especially, it may also be a promising detector of HCC‐related mutations.

#### 3.2.5 CRISPR‐Dx for Further Applications

Besides the representative CRISPR‐Dx platforms aforementioned, more and more new CRISPR‐Dx approaches have been reported. Timely, a CRISPR‐Dx platform to detect and supervise viruses, SARS‐CoV‐2 causing coronavirus disease 2019 (COVID‐19), has attracted wide discussion. Hampton *et al*. reported a Cas13‐developed combinatorial arrayed reactions for multiplexed evaluation of nucleic acid (CARMEN) assay.^[^ ^79^ ^]^ This new technique was proven using over 4500 tests on a large‐capacity microfluidic chip. The detailed process was that the amplified viral sample would be labeled via fluorescent dye in which CRISPR/Cas13 could identify the specific viral genetic sequence, resulting in a related color‐coded signal. For rapid detection at home or in small clinics, Liu and co‐workers developed All‐In‐One Dual CRISPR‐Cas12a (AIOD‐CRISPR) assay as a fast, ultrasensitive, and visual approach for SARS‐CoV‐2 detection.^[^ ^80^ ^]^ They validated the AIOD‐CRISPR assay to detect clinical swab samples in 20 min with a low‐cost hand warmer as an incubator.

CRISPR‐Dx is such a powerful toolbox with a variety of biomarkers for further diagnostic applications. Current CRISPR/Cas diagnostic platforms can detect viral infections (*e.g*., HPV and SARS‐CoV‐2), cancer markers (*e.g*., breast cancer markers), or other genetic signatures. With rapid development, great efforts would be transformed into molecular diagnostics for more diseases, including viral hepatitis and HCC discussed in this review. With the patient samples, such as cells, blood, sputum, urine, and feces, the CRISPR/Cas detection has the potential to achieve viral detection, cancer subtype classification, and so on in the fields of viral hepatitis and HCC (Figure 4B).

## 4 CRISPR Technology for Therapeutics of Viral Hepatitis and Hepatocellular Carcinoma

CRISPR/Cas system is an emerging approach for gene therapy in the liver.^[^ ^32b^ ^]^ For treating viral hepatitis and HCC, a safe, efficient, and specific CRISPR delivery system to ensure a functional Cas9/gRNA complex present into the target sites is the key. In addition, CRISPR/Cas‐based therapy in conjunction with other therapeutic strategies may enhance therapeutic efficacy. This section will focus on these aspects to discuss the applying prospects and potential use of CRISPR/Cas systems for treating viral hepatitis and HCC. Particularly, different delivery approaches for transfection of the CRISPR elements are highlighted here.

### 4.1 CRISPR Gene Editing to Target Viral Hepatitis and Hepatocellular Carcinoma

#### 4.1.1 CRISPR Technology in Viral Hepatitis Gene Therapy

Recently, CRISPR/Cas editing to human viral pathogens has made significant progress, especially hepatitis viruses like HBV and HCV.^[^ ^81^ ^]^ Conventionally, antiviral agents such as reverse transcriptase inhibitors (nucleoside or nucleotide analogs) and RNA interference (RNAi) technology were utilized to combat viruses in the liver. Different delivery approaches for transfection of the CRISPR elements are highlighted. The CRISPR/Cas technology has added to the armamentarium of therapeutic strategies with higher efficiency and specificity to treat viral hepatitis (**Table** **5**).

**Table 5:** CRISPR‐based therapeutic studies in viral hepatitis

Hepatitis viruses	Therapeutic targets	In vitro or in vivo models	Methods and vectors	Gene‐editing efficiency (%)	Therapeutic effects	Ref.
HBV1.2	P1(1292‐1314), XCp (1742‐1764)	Huh7 cells, mouse models with a hydrodynamic injection of 1.2× HBV plasmids	Human codon‐optimized Cas9 (hCas9) plasmid and sgRNA plasmid delivered by lipofectamine *(in vitro*) and hydrodynamic injection (HDI) (in vivo)	In vitro: 25.6% In vivo: about 5%	Decrease of cccDNA and rcDNA	^[^ ^159^ ^]^
HBV concentrated 100‐fold from the culture medium of HepAD38 cells	ENII/CP/X (2987‐3006; 3048–3067; 3062–3081), Pre‐C (2‐21)	HepG2 cells expressing sodium taurocholate co‐transporting polypeptide (NTCP)	CW‐Cas9 plasmid and sgRNA plasmid delivered by lentivirus (in vitro)	In vitro: over 60%	Eightfold HBV inhibition	^[^ ^24^ ^]^
HCV	HCV RNA	Huh‐7.5 cells	FnCas9/rgRNA plasmid delivered by lipofectamine (in vitro)	Unshown	Inhibition of HCV protein production	^[^ ^41^ ^]^
HBV1.3	X (1523‐1542; 1681–1700)	Huh7 cells, HepG2.2.15 cells, mouse models with a hydrodynamic injection of precccDNA plasmid	PX330 delivered by lipofectamine (in vitro) and HDI (in vivo)	In vitro: 44.2% (gRNA1) and 34.2% (gRNA2) In vivo: unshown	Inhibition of intracellular cccDNA (with >60% decrease) and viral replication	^[^ ^160^ ^]^
HBV 1.3	S1 (357‐376), X1 (1406‐1425)	HepG2 cells, HepG2.2.15 cells, HBV‐transgene (Tg) mice	hCas9 plasmid and sgRNA plasmid delivered by PEI (in vitro) and HDI (in vivo)	In vitro: unshown In vivo: over 50%	Over 50% decrease of HBsAg and 58–75% mutations in HBV DNA	^[^ ^84^ ^a]^
HBV 1.3	ORF S, core, polymerase, X	HepG2 cells, HepG2.2.15 cells, immunodeficient mice (NRG) with a hydrodynamic injection of 1.3x HBV plasmids	hCas9/sgRNA plasmid delivered by lentivirus (in vitro) and HDI (in vivo)	In vitro: over 60% In vivo: unshown	Decrease of both cccDNA and other HBV‐related parameters of expression and replication	^[^ ^161^ ^]^
HBV concentrated 100‐fold from the culture medium of HepAD38 cells or HepG2.2.15 cells	HBx2 (2871‐2893), HBx4 (2827‐2849)	HepG2 cells expressing NTCP	CW‐Cas9 plasmid and sgRNA plasmid delivered by lentivirus (in vitro)	In vitro: over 80%	90% cleavage of HBV DNA	^[^ ^93a^ ^]^
HCV	miR‐122 locus (hcr)	Huh‐7 cells	Cas9/sgRNA plasmid with the homologous recombination template pSSV9‐hcr‐donor‐shmiRHCV318 delivered by AAV (in vitro)	In vitro: nearly 30%	Expression of anti‐HCV shmiRNA after site‐specific integration, destroy of a subgenomic HCV replicon and a full‐length reporter virus	^[^ ^90^ ^]^
HBV1.05	DNA polymerase *κ* (POLK): sgPOLK‐1(5’‐CTTCTCCTTTGTGCTATCCA‐3’), sgPOLK‐2 (5’‐GATGATCTTCTGCTTAGGAT‐3’)	HepG2 cells expressing NTCP	CW‐Cas9 plasmid delivered by lipofectamine and sgRNA plasmid delivered by lentivirus (in vitro)	Unshown	Inhibition of rcDNA converting into cccDNA, a >50% decrease of cccDNA formation	^[^ ^120^ ^]^
HBV1.3	ORF S, core, polymerase, X: sgB1 (5’‐ GAGGTGAAGCGAAGTGCACA‐3’), sgB2 (5’‐ CCACCCAAGGCACAGCTTGG‐3’), sgB3 (5’‐ CGGGGAGTCCGCGTAAAGAG‐3’), sgB4 (5’‐ AAGCCACCCAAGGCACAGCT‐3’), sgB5 (5’‐ GAAGCGAAGTGCACACGGTC‐3’), sgB6 (5’‐ AGAAGATGAGGCATAGCAGC‐3’), sgB7 (5’‐ CAAGCCTCCAAGCTGTGCCT‐3’), sgB8 (5’‐ GGGGCGCACCTCTCTTTACG‐3’), sgB9 (5’‐ GGACTTCTCTCAATTTTCTA‐3’)	HepAD38 cells, mouse models with hydrodynamic injection of 1.3x HBV plasmids	Cas9 mRNA/sgRNA delivered by lipid‐like nanoparticle (LLN) (in vitro and in vivo)	Unshown	Induction of indels in the HBV DNA, decrease of all measurements of HBV viral loads	^[^ ^84b^ ^]^
HBV1.1	ORF S4 (368‐390), S5 (688‐710), XP (1257‐1278), CP‐BCP (1868‐1890), CP‐URR (1682‐1703)	HepG2.A64 (CCTCC C 201163) cells	PX459 delivered by lipofectamine (in vitro)	Unshown	Full eradication of HBV cccDNA and the full length of integrated HBV DNA	^[^ ^162^ ^]^
HBV1.2 and HBV1.3	ORF S (56‐75), P (1179‐1197), X (1575‐1595), C1 (1865‐1884; 2367–2386)	Huh‐7 cells, HepAD38 cells, HepG2 cells expressing NTCP, mouse models with a hydrodynamic injection of 1.2x HBV plasmids	PX458 delivered by lipofectamine (in vitro) and HDI (in vivo)	Unshown	Synergistic effect to inhibit HBV replication and destroy HBV genome	^[^ ^163^ ^]^
HBV1.3 and HBV1.2	Sa1 (252‐278), Sa2 (1377‐1403), Sa4(2378‐2405)	Huh7 cells, HepG2.2.15 cells, HepHepAD38 cells, mouse models with a hydrodynamic injection of 1.2x HBV plasmids	PX601 delivered by lipofectamine (in vitro), HDI and AAV (in vivo)	In vitro: 28.3% In vivo: unshown	Decrease of HBsAg, HBV DNA, and pgRNA	^[^ ^164^ ^]^
HBV1.3	HBV‐reverse transcriptase (HBV‐RT, 5’‐ TTCAGTTATATGGATGATG‐3’), P1 (5’‐ GTTTTGCTCGCAGCAGGTCT‐3’, XCp (5’‐ GGGGGAGGAGATTAGGTTAA‐3’)	HepG2.2.15 cells, HepG2 cells expressing NTCP	PX330 delivered by high‐capacity adenovirus (HCAdV) (in vitro)	In vitro: 37.4%	A decrease of HBV antigen production, the introduction of indels the HBV genome, degradation of cccDNA	^[^ ^83b^ ^]^
HBV1.28	ORF S, X, P, C: 21 gRNAs	HepG2 cells, HepG2.2.15 cells, HBV‐Tg mice	PX601 delivered by rAAV type 8 (in vitro and in vivo)	In vitro: unshown In vivo: 41.05%	Decrease of serum HBsAg, HBeAg levels, HBV DNA, and liver‐cell HBcAg	^[^ ^84c^ ^]^
HBV1.1 and HBV1.5	ORF S, X, P, C: 50 gRNAs with SpCas9, 6 gRNAs with NmCas9, 10 gRNAs with StCas9, 5 gRNAs with FnCas9	HepG2 cells	SpCas9‐EGFP, NmCas9, StCas9, or FnCas9/gRNA plasmid transfection via nucleofection (in vitro)	In vitro: over 85%	Inhibition of HBV replication up to 60%, degradation of over 90% HBV cccDNA	^[^ ^165^ ^]^
HBV1.2	gHBV1 (5’‐CAAGCCTCCAAGCTGTGCCT‐3’), gHBV2 (5’‐GGTTGCGTCAGCAAACACT‐3’)	HepAD38 cells, Huh7 cells	PX458 delivered by endogenous exosomes (in vitro)	Unshown	Inhibition of HBV replication	^[^ ^83a^ ^]^
HBV concentrated from the culture medium of HepAD38 cells	Human apolipoprotein E (apoE, 5’‐CACCGGCTTTTGGGATTACCTGCGC‐3’)	HepAD38 cells, HepG2 cells expressing NTCP	Cas9/sgRNA plasmid delivered by lipofectamine (in vitro)	Unshown	Over 90% reduction of HBV infection and over 80% decrease of HBV production	^[^ ^93b^ ^]^
HBV1.3	P (608‐630; 929–951; 930–952; 931–953; 1048–1070; 1074–1096; 1078–1100; 1328–1350; 1632–1654), ORF S (1053‐1075; 1054–1076; 1263–1285; 1305–1327; 1519–1541; 1521–1543; 1887–1909; 1888–1910; 1933–1955)	HepG2.2.15 cells, Huh‐7 cells, HepG2‐NTCP‐C4 cells	pLenti‐FNLS‐P2A‐Pur (BE3) and pLenti‐BE4Gam‐P2A‐Pur (BE4), sgRNA plasmid delivered by lipofectamine (in vitro)	In vitro: approximately or greater than 50%	Inhibition of HBV gene expression, inactivation of integrated HBV DNA and cccDNA	^[^ ^46^ ^]^
HBV concentrated from the culture medium of HepG2.2.15 cells	ORF S (crRNA: 5’‐ AGCTTGGAGGCTTGAACAGT‐3)’	HepG2‐NTCP‐30 cells	Cas9/sgRNA RNP and ss‐ON complex delivered by LNP in a microfluidic device (in vitro)	Unshown	Decrease of HBV DNA and cccDNA with 60% and 80%, respectively	^[^ ^97^ ^]^

**Figure 5:**
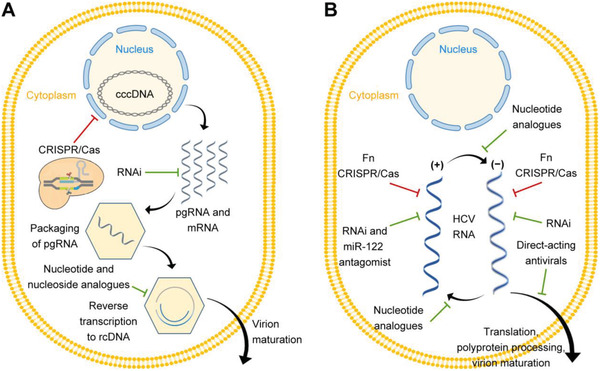
Schematic representation of viral hepatitis and therapeutic strategies. A) HBV viral infections and contemporary therapeutic strategies. CRISPR/Cas technology mainly targets HBV cccDNA for therapy. B) HCV viral infections and contemporary therapeutic strategies. CRISPR/Cas technology mainly targets HCV RNA for treatments with the FnCas9 system. Adapted with permission.^[^ ^81^, ^142^ ^]^ Copyright 2018, Elsevier.

For HBV‐induced viral hepatitis, cccDNA is the key therapeutic target (**Figure** **5**A). It contains four long open reading frameworks (ORFs): surface‐ (S), core‐ (C), polymerase‐ (P), and X protein‐encoding segments. After translation, seven main proteins are highly involved in viral replication. Extracellular HBV DNA is a relaxed circular dsDNA (rcDNA) generated by the reverse transcription of pregenomic RNA (pgRNA). With the help of viral proteins and host cytokines, intracellular cccDNA is formed via the repair of pgRNA. The episomal cccDNA can act as a template and guarantee viral production and sustainable infection in the HBV life cycle.^[^ ^82^ ^]^ Therefore, targeting the HBV cccDNA and its intermediates can effectively suppress HBV and consequent tumorigenesis. Several CRISPR/Cas systems were reported for HBV‐specific gene disruption to target the conserved regions of cccDNA in vitro^[^ ^24^, ^46^, ^83^ ^]^ and in vivo,^[^ ^24^, ^84^ ^]^ illustrating the bright future of HBV elimination via CRISPR/Cas gene editing.

Unlike HBV, HCV replicates in the cytoplasm, for which the key to preventing HCV‐infected hepatitis is to tackle the virus RNAs (Figure 5B). The insertion of HCV RNA in hepatocytes allows continuous viral production. To solve the dilemma to radically remove viral infections, CRISPR/Cas9 systems to silence HCV RNA have emerged. Price *et al*. employed FnCas9 with RNA‐targeting guide RNA (rgRNA)to target HCV mRNA.^[^ ^41^ ^]^ The FnCas9 system showed over 50% reduction of HCV protein expression.

#### 4.1.2 CRISPR Technology in Hepatocellular Carcinoma Gene Therapy

HCC, one of the most common cancer types, is still challenging to be effectively treated due to its extraordinary genetic heterogeneity. Nowadays, CRISPR technology has emerged as a promising therapeutic approach to enhance anti‐HCC efficacy (**Table** **6**).

**Table 6:** CRISPR‐based HCC therapeutic strategies

Therapeutic targets	Functions	In vitro or in vivo models	gRNA design	CRISPR manipulation	Methods and vectors	Gene‐editing efficiency (%)	Therapeutic effects	Ref.
ZIC2	A transcription factor (TF) for self‐renewal maintenance of liver cancer stem cells (CSCs)	Hep3B cells, mouse models with tumor xenograft	5’‐CCATCACCACTCCGCCGCGG‐3’, 5’‐TTCACGGTCCTGCATCTCGG‐3’	Knockout	Cas9/sgRNA plasmid delivered by lentivirus (in vitro) and CRISPR‐engineered Hep3B cells delivered by e subcutaneous injection (in vivo)	Unshown	Inhibition of self‐renewal of liver cancer stem cells (CSCs) and tumor propagation	^[^ ^25^ ^]^
Aspartate *β*‐hydroxylase (ASPH)	A transmembrane protein member in *α*‐ketoglutarate‐dependent dioxygenase family	HepG2 cells	5’‐ATGGAGGACACAAGAATGGG‐3’, 5’‐TAAACAGAGACAAAGCATGG‐3’, 5’‐CCTAGTACAAAATACGTGACGTAGAA‐3’	Knockout	Cas9/sgRNA plasmid delivered by human immunodeficiency viruses (HIV) (in vitro)	Unshown	Inhibition of tumor growth and induction of tumor cell senescence	^[^ ^166^ ^]^
BAX and BCL2	Related to the sensitivity of cells to apoptosis	HepG2 cells	BAX: sgRNA38, 5’‐GAGAACAGGGTACGATAACCGTTTTAGAGCTAGAAATAGCAAGTTAAAATAAGGCTAGTCCGTTCGTACACCATCAGGGTACGTCGTACCCTGTTCTCAGAGCGGAAGCGTGCTGGGCTCCGAACAGCGGAAGGTGGTTCGAAGCTGGGGCTTTGGACATAAGAGAACAGGTTTTTT‐3’; BCL2: sgRNA39, 5’‐ GACGGGACCAAACCTCCCGAGTTTTAGAGCTAGAAATAGCAAGTTAAAATAAGGCTAGTCCGTTGGTTTAATCAGAGTAGAGGAGCTGACTCCTTTGGTTGGACTAAGGTTTGGTCCCGTCAGAGCGGAAGCGTGCTGGGCTCCGAACAGCGGAAGGTGGTTCGAAGCTGGGGCTTTGGACATAAGACGGGACCTTTTTT‐3’	CRISPRi for BCL and CRISPRa for BAX	MS2‐dCas9+sgRNA38 and Rev‐dCas9‐VP64+sgRNA39 plasmid delivered by lipofectamine (in vitro)	In vitro: up to 8‐fold activation and 80% repression	Induction of tumor cell apoptosis	^[^ ^167^ ^]^
CXC chemokine receptor 4 (CXCR4)	A specific receptor of chemokine stromal cell‐derived factor‐1 (CXCL12) with a strong chemotaxis effect on lymphocytes	HepG2 cells, mouse models with tumor xenograft	5’‐CACCGGGCAATGGATTGGTCATCC‐3’	Knockout	Cas9/sgRNA plasmid delivered by lipofectamine (in vitro) and CRISPR‐engineered HepG2 cells delivered by e subcutaneous injection (in vivo)	In vitro: 29.5%	Nearly 50% CXCR4 downregulation, inhibition of tumor proliferation, migration, invasion the malignancy, reversion of epithelial‐mesenchymal transition (EMT), increased chemosensitivity to the antitumor drug cisplatin	^[^ ^168^ ^]^
Euchromatic histone‐lysine *N*‐methyltransferase 2 (EHMT2), also known as G9a	A lysine methyltransferase to di‐methylate lysine 9 of histone H3 (H3K9me2)	BEL‐7402 cells, SMMC‐7721 cells, THLE‐3 cells, mouse models with orthotopic tumor implantation	5’‐GGGTCACTTCTCCTGAACGC‐3’, 5’‐GGTCACTTCTCCTGAACGCC‐3’	Knockout	PX459 delivered by lentivirus (in vitro) and CRISPR‐engineered BEL‐7402 cells delivered by e subcutaneous injection (in vivo)	Unshown	Inhibition of the proliferation and migration of HCC cells in vitro, inhibition of HCC tumorigenicity in vivo	^[^ ^169^ ^]^
Granulin (GRN)	A potent pluripotent mitogen and growth factor maintaining self‐renewal of liver CSCs	Hep3B cells	5’‐TAGAGATGATAGCGCGTGTCTGG‐3’, 5’‐GGCGCCTGCAGGATGGGTTAAGG‐3’ 5’‐TTGGAGAATCATGTGACGTCGG‐3’ 5’‐GATCCCTAGAAATGGGGTGTGG‐3’	CRISPRi	dCas9‐suppressor plasmid and gRNA plasmid delivered by lipofectamine (in vitro)	In vitro: about 80%	Inhibition of proliferation and invasion up to fourfold	^[^ ^43^ ^]^
Glutaminase 1 (GLS1)	An enzyme converting glutamine to glutamate, which is highly expressed in HCC	LO2 cells, SMMC‐772 1cells, HCCLM3 cells, Hep3B cells, mouse models with tumor xenograft	Unshown	Knockout	Cas9/sgRNA plasmid delivered by lentivirus (in vitro) and CRISPR‐engineered HCCLM3 and SMMC‐7721 cells delivered by e subcutaneous injection (in vivo)	Unshown	Decrease of stemness‐related genes expressing, inhibition of CSC properties, and tumorigenicity	^[^ ^170^ ^]^
CCAAT/enhancer‐binding protein‐beta (C/EBP*β*)	A recurrent hypomethylated enhancer related to poorer HCC prognosis	LO2 cells, BEL‐7404 cells, Hep3B cells, HepG2 cells, Huh7 cells, PLC5 cells, SK‐Hep1 cells, mouse models with tumor xenograft	5’‐CACACACACAGGGCCACCGA‐3’	Knockout	Cas9/sgRNA plasmid delivered by jetPRIME (in vitro) and CRISPR‐engineered HCC cell lines delivered by e subcutaneous injection (in vivo)	Unshown	Inhibition of driver oncogenes and tumorigenicity	^[^ ^171^ ^]^
Nuclear receptor binding SET domain‐containing protein 1 (NSD1)	Involving in tumorigenesis via regulating Wnt/*β*‐catenin signaling pathway	Huh7 cells, Hep3B cells, SMMC‐7721 cells, HepG2 cells, SK‐Hep1 cells, mouse models with tumor xenograft	5’‐TTGGATTGACCATTACCGAA‐3’, 5’‐TGGATTGACCATTACCGAAA‐3’, 5’‐GCAAGTGCTGTAGGACCACC‐3’	Knockout	Cas9/sgRNA plasmid delivered by lentivirus (in vitro) and CRISPR‐engineered HCC cell lines delivered by e subcutaneous injection (in vivo)	Unshown	Inhibition of tumor proliferation, migration, invasion	^[^ ^172^ ^]^
Zinc‐finger protein 384 (ZNF 384)	Promoting tumor growth by upregulating Cyclin D1 expression	Huh7 cells	5’‐CACCGGCCTCAGTGTCCCTGCCCTC‐3’, 5’‐CACCGGCCAGAGAAGGGCTGTGGTC‐3’	Knockout	wPGL3 plasmid delivered by lentivirus (in vitro)	Unshown	Inhibition of tumor proliferation via inhibition of Cyclin D1	^[^ ^173^ ^]^
lncRNA‐RP11‐156p1.3	Belonging to HCC‐associated lncRNA network	HepG2 cells	5’‐GCCGGGGAGCAGGGTGCGCCGGG‐3’, 5’‐ACGACGACGTAGGATGCGCCAAA‐3’	Knockout	RNP delivered by CRISPRMAX	Unshown	Significant decrease of cell viability, TNF *α* and NF*κβ* protein levels	^[^ ^174^ ^]^
Epidermal growth factor receptor (EGFR)	A transmembrane receptor‐associated with the growth and proliferation of HCC	HepG2 and Huh7 cells, H22 cells‐bearing mice	5’‐GCATGGCGCCGTGCGGTTCA‐3’, 5’‐AGTAACAAGCTCACGCAGTT‐3’	Knockout and combing with sorafenib	PX458 delivered by an aptamer‐coated hollow mesoporous silica nanoparticle (in vitro and in vivo)	In vitro: 66.3% In vivo: unshown	Efficient in vitro EGFR‐editing and in vivo gene therapy for tumor inhibition as well as good synergistic drug therapy	^[^ ^110^ ^]^
Survivin (BIRC5)	Directly mediating tumor recurrence and metastasis	BEL‐7402 cells, BEL‐7402 cells‐bearing mice	5’‐TCTTGAATGTAGAGATGCGG‐3’	Knockout and combing with sorafenib	Cas9/sgRNA plasmid delivered by a lactose‐derived branched cationic biopolymer (LBP) (in vitro and in vivo)	In vitro: 21.3% In vivo: 26.4%	Efficient in vitro editing and in vivo HCC therapy	^[^ ^104^ ^]^
WNT10B	A member of the Wnt family encoding secreted proteins	HepG2 cells and HepG2 cells‐bearing mice	5’‐TCTTGGTTCCCAGGGCTCTA‐3’, 5′‐ GCCTCCGCTCAGCTTAATCT‐3’	Knockout	RNP delivered by cell‐selective extracellular vesicle (in vitro, *ex vivo*, and in vivo)	In vitro: about 30% *Ex vivo*: unshown In vivo: unshown	Decreased the protein expression of WNT10B and tumor inhibition in vitro, *ex vivo*, and in vivo	^[^ ^112^ ^]^

Typically, the CRISPR/Cas systems have been utilized to treat HCC from two directions through editing: 1) the direct targets or 2) an indirect site to reverse the HCC progression. For the direct targeting, HCC‐associated genes, including oncogenes and TSGs are the direct therapeutic targets. For indirect strategies, CRISPR‐associated gene manipulations can synergize the efficacy in conjunction with antitumor drugs, immunotherapy, and other therapeutic agents or modifications.^[^ ^85^ ^]^ For instance, a study showed that CRISPR/Cas‐induced ERK2 kinases inhibition enhanced the response of HCC cell lines to sorafenib, a clinically recommended drug as the multi‐kinase inhibitor to treat HCC.^[^ ^86^ ^]^ Additionally, functional screens identified phosphoglycerate dehydrogenase (PHGDH), cyclin‐dependent kinase 5 (CDK5), and CDK12 could be credible targets of CRISPR‐mediated inhibition to synergize the antitumor effects of sorafenib.^[^ ^85b–d^ ^]^ Besides sorafenib, THZ1 (CDK7 oncogene inhibitors), metformin, and others are potential candidates in conjunction with CRISPR technology for HCC therapy.^[^ ^87^ ^]^

### 4.2 Nanomedicine for CRISPR Delivery

Delivery methods of CRISPR elements are generally both local and systemic administration approaches, and the latter are discussed in more details in the following sections. For the therapy of viral hepatitis and HCC, CRISPR delivery systems are the key to achieving gene editing and therapeutic efficacy. Reported works have demonstrated the possibility of delivering three different forms of the CRISPR/Cas system: highly negative‐charged plasmid and CRISPR/Cas‐encoding mRNA with gRNA, RNP complex with the integration of Cas9 protein (theoretical charge about +22 mV), and sgRNA (nearly 100 anionic phosphate groups).^[^ ^4^, ^54^, ^88^ ^]^

**Figure 6:**
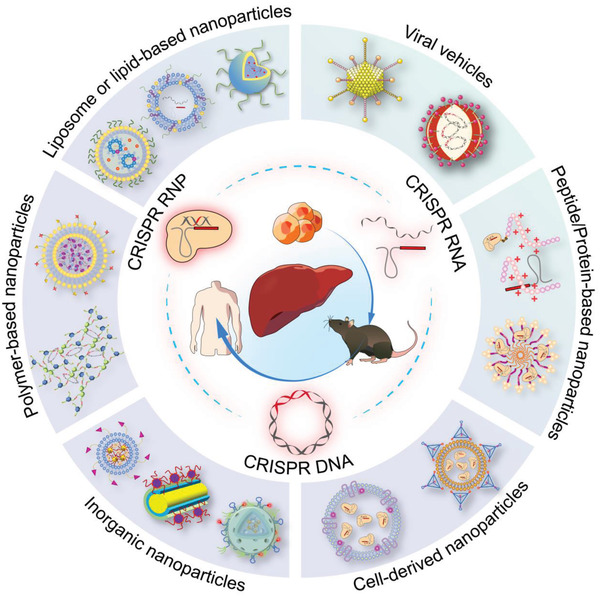
Emerging and potential CRISPR/Cas liver delivery via viral and non‐viral systems. There are three formats of CRISPR/Cas systems: plasmid DNA, Cas mRNA/gRNA, and RNP. They could be packaged into delivery vectors for in vitro, in vivo, and further human clinical applications of liver‐targeted gene therapy.

To date, the field has explored different delivery methods, including viral, non‐viral vectors, and physical delivery. Physical delivery strategies, particularly the electroporation method, can directly deliver gene constructs to target sites and have been applied in CRISPR‐engineered CAR‐T/NK cell therapy.^[^ ^88d^ ^]^ However, physical strategies are still limited by untenable cell function and difficulty to apply in vivo.^[^ ^88a^ ^]^ This section will focus on the viral and non‐viral CRISPR delivery systems (**Figure** **6**), followed by the perspectives on the current and the future directions of CRISPR/Cas nanomedicine in viral hepatitis and HCC.

**Figure 7:**
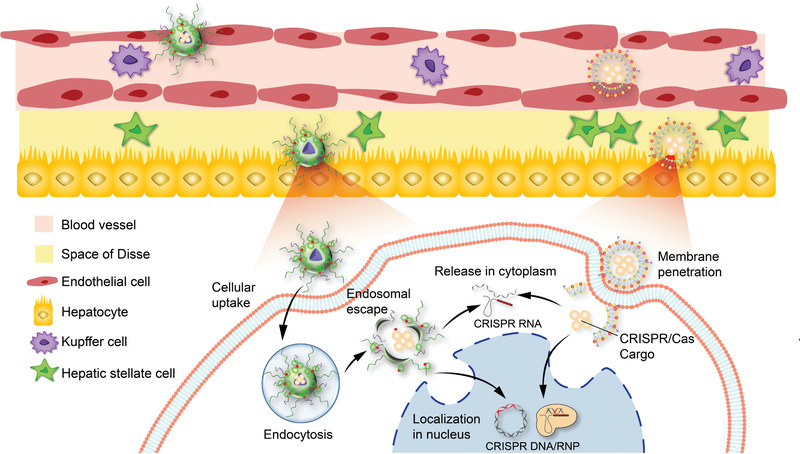
Non‐viral delivery of CRISPR/Cas systems to the liver. In the extracellular environment, nanoparticles with cargos go across the blood circulation and space of Disse to reach the sites of hepatocytes, in which they would be degraded or cleared via serum nucleases and immune cells. While in the intracellular microenvironment, efficient cell uptake generally by endocytosis or membrane fusion, on‐time release, and correct translocation without degradation are necessary.

Delivery vectors loaded with CRISPR cargos (plasmid DNA, mRNA/gRNA, and RNP) for liver delivery should successively pass through the blood circulation and vessels, space of Disse, and hepatocytes.^[^ ^89^ ^]^ Here, hepatitis virus‐infected or HCC‐developed hepatocytes should be the target cells for CRISPR/Cas therapeutic editing.^[^ ^89^ ^]^ For the in vivo route, therapeutic applications of CRISPR systems should overcome the following delivery barriers: 1) the large size of CRISPR/Cas cargos; 2) the limited biological stability via degradation by nucleases present in physiological fluids; 3) the restriction of crossing cell membranes with the hydrophilic characteristics and highly negative charge; 4) the likely degradation of endosomes and lysosomes even after cell uptake. Therefore, the CRISPR/Cas delivery system should be well‐protected and stable outside the target site, achieve efficient cellular internalization, and successfully release the carrier for therapeutic gene editing (**Figure** **7**).

#### 4.2.1 Viral Vectors

Viral vectors are well‐characterized delivery approaches for CRISPR/Cas gene editing. They have been widely used to deliver CRISPR/Cas systems to the liver.^[^ ^24^, ^46^, ^83^, ^90^ ^]^ Thus, development of viral vectors is quite active in the field for therapeutic editing in viral hepatitis and HCC.

Adenovirus and adeno‐associated virus (AAV)‐mediated CRISPR vectors are the most common types for viral CRISPR delivery. For example, Schiwon *et al*. designed a high‐capacity adenovirus to deliver multiplexed CRISPR/Cas9 systems with gRNAs to target the HBV genome and the cccDNA.^[^ ^83b^ ^]^ This treatment resulted in 70% cccDNA inhibition of chronic HBV infection. Furthermore, Luo and co‐workers designed an adenovirus vector encoding the CRISPR/Cas system to target the fusion gene for HCC therapy. They applied the gRNA to target the fusions in breakpoints of TMEM135‐CCDC67 and MAN2A1‐FER, and therefore achieved high‐specific antitumor effects in vitro and 30% shrinking of the tumor volumes in vivo.

Compared with adenoviruses, AAV is a single‐stranded DNA virus that the endogenous viral encoded arrays are removed and can deliver transgene up to 4.7 kb. AAV‐mediated CRISPR delivery reduces pathogenicity and possesses inherent tissue orientation.^[^ ^10^, ^91^ ^]^ Lately, Song and co‐workers used AAV‐delivered CRISPR/Cas9 to treat chronic HBV hepatitis.^[^ ^84c^ ^]^ This system resulted in significant inhibition of HBV in vitro and an efficient reduction of HBV‐related measurements in vivo during 58 days of continuous observation. Besides, Senis *et al*. used AAV‐delivered CRISPR/Cas9 to target miR‐122 locus (hcr) and integrate an anti‐HCV shmiRNA (an RNAi hairpin embedded in micro‐RNA).^[^ ^90^ ^]^ This combined therapy with CRISPR/Cas and RNAi was confirmed in vitro and reported ≈30% HDR‐induced editing as well as 10‐ to 100‐fold HCV reduction in viral replication.

Retroviruses and lentiviruses are also commonly used vectors.^[^ ^92^ ^]^ These vectors have been widely used to transduce CRISPR/Cas9 elements to treat viral hepatitis and HCC.^[^ ^24^, ^46^, ^93^ ^]^ Promisingly, to solve the problem of lentivirus‐mediated long‐lasting expression of CRISPR/Cas9 machinery, a report developed a lentivirus‐like bio‐nanoparticle (LVLP) delivery system for CRISPR mRNA in a “hit‐and‐run” manner for safe editing.^[^ ^92^ ^]^ The SaCas9 mRNA was efficiently packaged in LVLP via specific interaction between MS2 (an RNA aptamer) and MS2‐binding protein termed MCP. The delivery system retained the transduction efficiency of lentiviral vectors and performed transient editing expression within 24 h.

However, certain limitations and concerns of viral delivery have hindered further developments. First, some viral vectors, such as AAVs, have limited packaging capacity.^[^ ^4^ ^]^ Second, potential viral integration in the genome may lead to carcinogenesis. Third, the immunogenicity of the viruses may trigger pre‐existing and adaptive immune responses, thereby increasing liver toxicity.^[^ ^94^ ^]^ These concerns should be considered and addressed for virus‐delivered gene therapy.

#### 4.2.2 Non‐Viral Vectors

Compared with viral delivery, non‐viral delivery is safer as its transient expression nature, and it has been routinely used for gene delivery. Multiple non‐viral vectors are under development, and these may have better potential for translation. Several extracellular and intracellular barriers can compromise the delivery efficiency of non‐viral vectors. In general, the CRISPR delivery system with optimized dose should in time travel through the blood vessels and interstitial space without clearance and degradation of the body's protective mechanisms, then go across the membrane of the target cell types.^[^ ^88c^ ^]^ Inside the target hepatocytes in the liver, the delivery systems entered through micropinocytosis or endocytosis should escape from endosomal compartment into the cytoplasm (RNA format) and further into the nucleus (DNA and RNP format).^[^ ^95^ ^]^

##### Liposome or Lipid‐Based Nanoparticles

Liposome or lipid‐based nanoparticles with hydrophobic tails and hydrophilic head groups are the most widely used vectors in gene delivery, and there are many available products in the market.^[^ ^89^ ^]^ With relatively mature technology, lipid‐based vectors can be designed to diminish off‐target effects and adverse immune reactions, and thus they provide a safe condition to the CRISPR/Cas system to realize ideal gene‐editing efficiency.^[^ ^96^ ^]^

**Figure 8:**
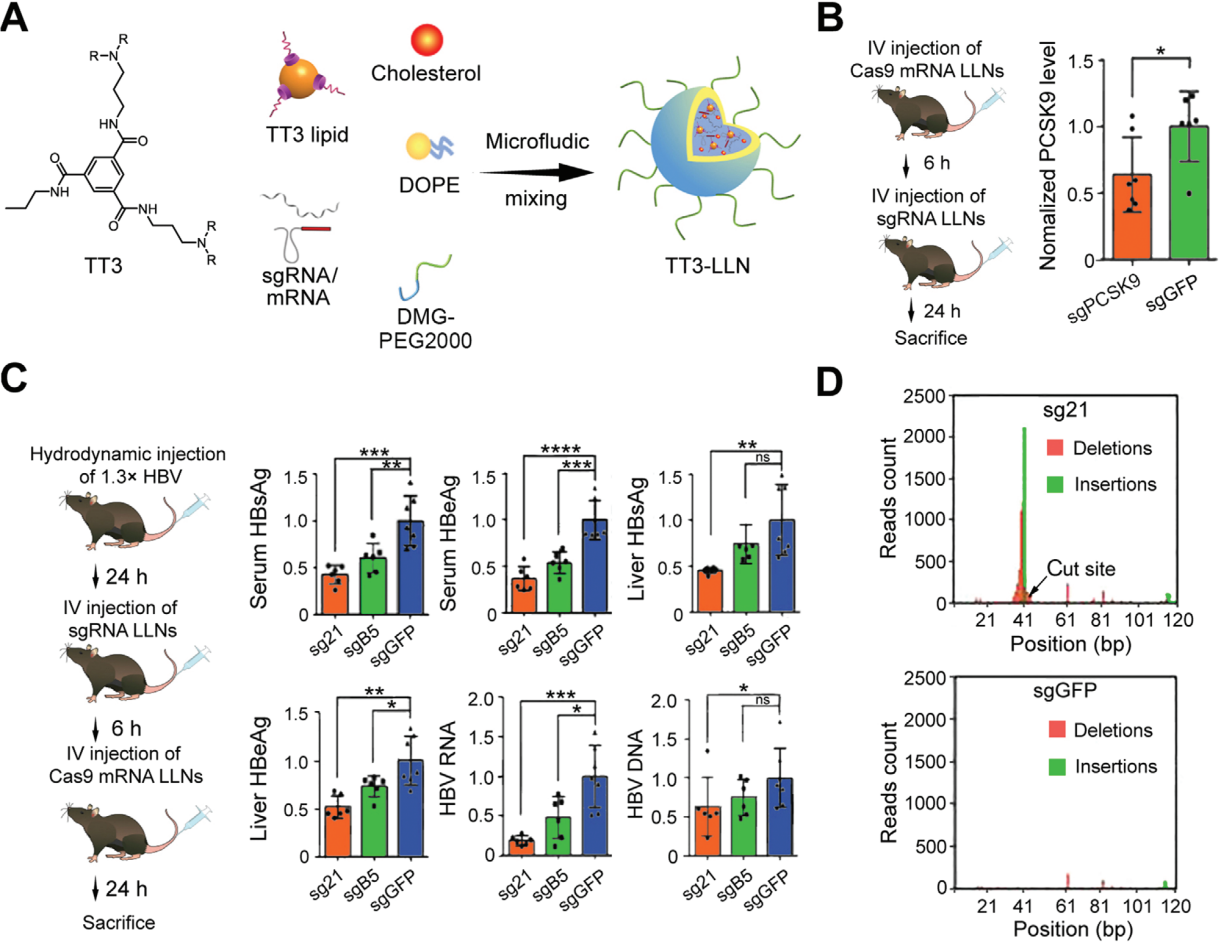
Lipid‐like nanoparticle (LLN) to deliver CRISPR/Cas9 component‐targeting PCSK9 gene and HBV DNA. A) The chemical constitution of TT3 and brief synthetic process of TT3‐LLNs. B) LLNs‐delivered CRISPR/Cas9 complex for PCSK9 gene knockout. *p < 0.05 (two‐tailed *t*‐test). C) HBV therapeutic efficiency of CRISPR/Cas9 delivered by LLNs. sg21 and sgB5 are both sgRNAs targeting the HBV cccDNA. *p < 0.05, **p < 0.01, ***p < 0.001, ****p < 0.0001 (two‐tailed *t*‐test). ns: *p* > 0.05. D) Deep sequencing analysis of TT3 LLN‐mediated gene editing. Reproduced with permission.^[^ ^84b^ ^]^ Copyright 2017, Springer Nature.

Increasing lipid‐delivered CRISPR/Cas systems have been developed for liver applications. Tan and co‐workers pioneered the well‐designed TT3 lipid‐like nanoparticle (LLN) to deliver the Cas9 mRNA/sgRNA therapeutic complex (**Figure** **8**A).^[^ ^84b^ ^]^ First, they injected Cas9 mRNA LLN to target the PCSK9 gene and achieve significant gene knockout (Figure 8B). They then turned to disrupt virus infection for HBV therapy, demonstrating nearly 50% anti‐HBV effects (Figure 8C) and high editing performance (Figure 8D).

In 2021, the team of Harashima used a mixer‐equipped microfluidic device to synthesize a lipid nanoparticle (LNP) for CRISPR RNP delivery and applied this system to exhibit therapeutic editing in HBV‐infected hepatitis.^[^ ^97^ ^]^ They prioritized the CRISPR cargo with an additional single‐stranded oligonucleotide (ssON) to gain more negative charges. After that, they mixed the RNP‐ssON complex and the prepared lipids (PEG lipid, cholesterol, phospholipid, pH‐sensitive cationic lipid) in the microfluidic device to establish cargo‐loaded LNP by electrostatic interaction. The LNP was identified to exhibit robust gene editing in HBV‐infected HepG2 cells and significantly inhibit HBV DNA and cccDNA to ≈60% and 80%, respectively.

**Figure 9:**
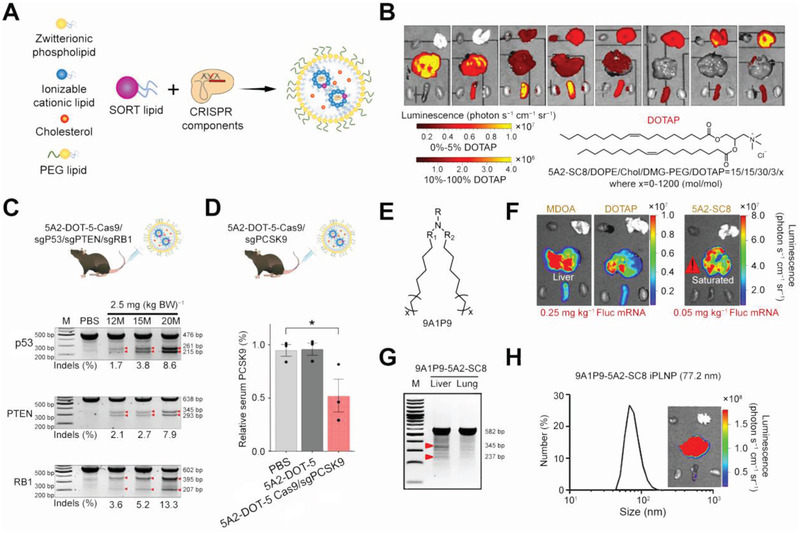
Lipid‐based selective organ‐targeting (SORT) strategy for liver‐targeting CRISPR delivery. A) SORT lipid was added to traditional lipids to deliver CRISPR components. B) SORT‐based LNPs with different ratios of SORT lipid in total lipids impacted the change of luciferase protein expression in the liver. Reproduced with permission.^[^ ^98a^ ^]^ Copyright 2020, Springer Nature. C) SORT‐mediated liver‐specific delivery for multiple targeting of p53, PTEN, and RB1 genes. D) SORT‐mediated liver‐specific delivery for therapeutic PCSK9 gene editing. *p < 0.05 (one‐way ANOVA). Reproduced with permission.^[^ ^98b^ ^]^ Copyright 2020, Springer Nature. E) Chemical structure of 9A1P9, optimized iPho. F) Fluorescence mRNA expression by 9A1P9 iPLNP with ionizable cationic helper lipids MDOA, DOTAP, and 5A2‐SC8. G) T7E1 assay of 9A1P9‐5A2‐SC8 encapsulating Cas9 mRNA/sgPTEN. H) Size and delivered fluorescence mRNA expression of 9A1P9‐5A2‐SC8 iPLNPs. Reproduced with permission.^[^ ^99^ ^]^ Copyright 2021, Springer Nature.

Especially, liver‐targeting strategies in lipid‐based delivery systems have been in fast development. Siegwart's group reported the selective organ‐targeting (SORT) strategy for LNP‐delivered CRISPR components.^[^ ^98^ ^]^ They synthesized LNPs with traditional lipids (zwitterionic phospholipid, ionizable cationic lipid, cholesterol, polyethylene glycol (PEG) lipid), and additional SORT lipid, such as DOTAP (permanent cationic lipid) (**Figure** **9**A). The systematically engineered LNPs could be liver targeted by adjusting the ratio of DOTAP from 0% to 5% (Figure 9B). The mouse model successfully delivered CRISPR components used SORT‐based LNPs to deliver CRISPR components into the liver. They achieved multiple gene targeting of p53, PTEN, and RB1 genes (Figure 9C) and further therapeutic editing of PCSK9 knockout (Figure 9D). More recently, they reported a series of synthesized ionizable phospholipids with high membrane destabilizing capacity (iPhos) to deliver CRISPR mRNA or RNP.^[^ ^99^ ^]^ The iPhos‐based nanoparticle (iPLNP) included a broad range of phospholipid components and could perform SORT delivery via the improvement of phospholipid structures and the changes of helper lipids. For liver targeting, they found iPhos with 9–12 carbon chains or combing the selected iPho, 9A1P9 (Figure 9E) with ionizable cationic lipids of MDOA and 1,2‐dioleoyl‐3‐dimethylammonium‐propane (DODAP) or 5A2‐SC8 (Figure 9F) could achieve liver‐specific delivery. iPLNP for co‐delivery of Cas9 mRNA/sgRNA‐targeting PTEN gene was verified to achieve in vivo liver‐targeting therapeutic editing at nearly 20% indels (Figure 9G,H).

Liposome or lipid‐based nanoparticles have been commonly used as delivery platforms. The efficient encapsulation and the affinity of cellular membranes make these systems preferable for CRISPR delivery.^[^ ^88c^ ^]^ Future advancements may focus on physicochemical parameters, such as rigidity, composition, size, and molecular structure. Promisingly, these vectors have potential as a protective and functional shell to combine merits with other biomaterials as multifunctional platforms encapsulating gene‐editing cargo for liver‐related applications.

##### Polymer‐Based Nanoparticles

Polymer vectors, usually containing cationic groups, are also widely studied to deliver CRISPR/Cas components. These vectors’ cationic nature allows the electrostatic complexation and protection of negative‐charged nucleic acid cargoes and enhanced cell uptake.^[^ ^100^ ^]^ The physicochemical properties (*e.g*., charge density, pKa, molecular weight), branching extents, biodegradability, and surface modifications can significantly impact the delivery capacity of polymeric vectors.^[^ ^100^, ^101^ ^]^ Based on the format of CRISPR cargo, the relational design of polymeric nanocarriers is critical for effective delivery.^[^ ^102^ ^]^

**Figure 10:**
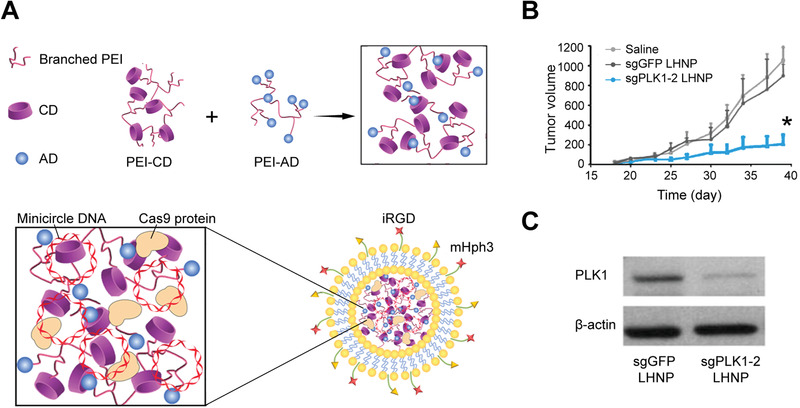
Liposome‐templated hydrogel nanoparticles (LHNPs) to deliver minicircle DNA coupled with Cas9 nuclease‐targeting PLK1 gene for tumor inhibition. A) Preparation of LHNPs. Cyclodextrin‐adamantine (CD‐AD)‐regulated host−guest interaction in breached PEI, as the core for co‐delivery of Cas9 protein and minicircle DNA. The DOTAP liposome modified with iRGD and mHph3 acted as shell. B) Antitumor effects of LHNP‐delivered CRISPR/Cas9. *p < 0.05 (unpaired t‐test). C) Regulation of PLK1 via CRISPR/Cas9 gene editing. Reproduced with permission.^[^ ^103^ ^]^ Copyright 2017, Wiley‐VCH.

Polyethyleneimine (PEI), containing amine groups and repeated two‐carbon (CH_2_−CH_2_), is the most used polymer‐based vector for CRISPR encapsulation. Combined with liposome materials, Chen *et al*. synthesized a core–shell 1,2‐dioleoyl‐3‐trimethylammoniumpropane (DOTAP) liposome‐PEI hydrogel nanoparticle (LHNP) to achieve efficient CRISPR delivery.^[^ ^103^ ^]^ This LHNP contained a PEI hydrogel‐core and a cationic lipid DOTAP‐shell modified with iRGD and mHph3 peptide ligands. Then, the nanoparticle was used to encapsulate both the Cas9 protein and minicircle DNA that encoded an sgRNA targeting the PLK1 gene (**Figure** **10**A). This strategy could disrupt the PLK1 gene in U87MG cells‐bearing mice, exhibiting antitumor effects with significant reduction of tumor volume (Figure 10B) and over 50% downregulation of PLK1 (Figure 10C). As PLK1 gene editing is a broad‐spectrum antitumor way, this strategy is promising to be transformed into HCC treatment.

**Figure 11:**
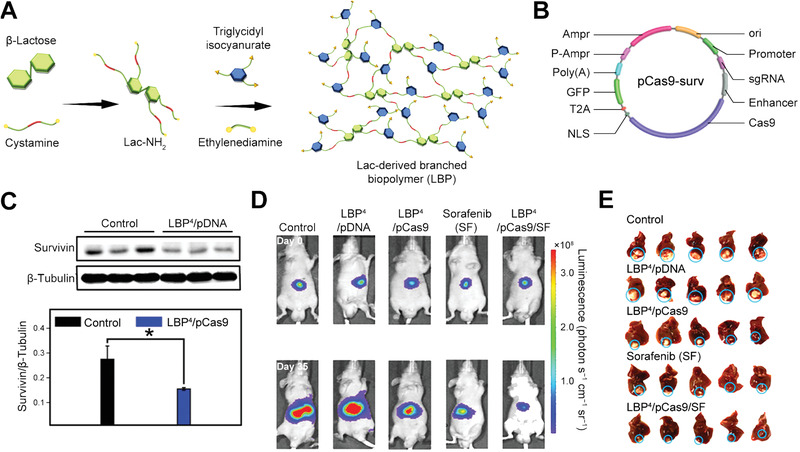
Lactose‐derived branched biopolymer (LBP) for delivery of CRISPR plasmid Cas9/sgRNA‐targeting survivin gene to treat orthotopic HCC. A) Preparation of LBP via a one‐pot ring‐opening reaction. B) Structure of CRISPR/Cas9 plasmid‐targeting surviving oncogene. C) Western blot analysis and corresponding statistical analysis of LBP/pCas9 and control groups in BEL‐7402 cells. *p < 0.05 (two‐tailed *t*‐test). D) Bioluminescence images of orthotopic HCC mouse models. E) Liver images of each treatment group. Reproduced with permission.^[^ ^104^ ^]^ Copyright 2020, Wiley‐VCH.

Polymer‐based nanoparticles can perform liver‐targeting capacity with modification strategies like targeting ligands. For example, Qi *et al*. developed a lactose‐derived branched cationic biopolymer (LBP) for HCC targeting via the binding of lactose and asialoglycoprotein receptor (ASGPR), which is highly expressed on the surface (**Figure** **11**A).^[^ ^104^ ^]^ With cationic polymers and reducible disulfide linkages, LBP was applied to deliver plasmid Cas9/sgRNA‐targeting survivin gene (Figure 11B) and showed excellent in vitro gene editing with 21.3% mutation (Figure 11C). In mouse models with orthotopic HCC xenograft, the LBP‐delivered CRISPR system performed 26.4% editing efficiency at survivin oncogene, thus inhibiting tumor growth and metastasis (Figure 11D,E).

There is a wide range of functional polymers available for delivering gene‐editing tools. Their physicochemical characteristics are tunable and thus feasible for applications at the cell and/or animal level. However, delivery with polymer nanoparticles usually encounters cytotoxicity, especially when the monomers are not removed completely.^[^ ^54^, ^88^ ^]^

##### Inorganic Nanoparticles

**Figure 12:**
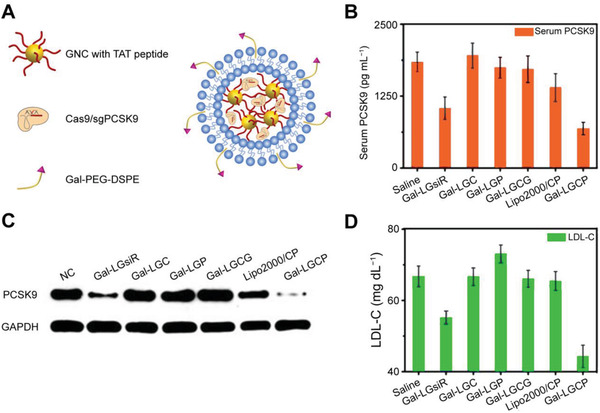
Gold nanoclusters complexed with cationic TAT peptide and a galactose lipid for CRISPR delivery to knock out PCSK9 gene in the liver. A) Scheme of gold‐based liver‐targeting CRISPR/Cas9 delivery. B) Quantitative analysis of serum PCSK9 levels. C) Western blot analysis of protein expression. Gal‐LGsiR, gal‐conjugated PEG lipid/TAT‐GNCs/siRNA. Gal‐LGC, gal‐conjugated PEG lipid/TAT‐GNCs/Cas9. Gal‐LGP, Gal‐conjugated PEG lipid/TAT‐GNCs/sgPCSK9. Gal‐LGCG, gal‐conjugated PEG lipid/TAT‐GNCs/Cas9/sgGFP), Lipo2000/CP, Lipo2000/Cas9/sgPCSK9. Gal‐LGCP, gal‐conjugated PEG lipid/TAT‐GNCs/Cas9/sgPCSK9. NC, negative control treated with PBS. D) Analysis of LDL‐C levels. Reproduced with permission.^[^ ^105^ ^]^ Copyright 2019, Wiley‐VCH.

Inorganic nanoparticles, especially gold nanoparticles and their derivatives, are being tested for CRISPR delivery in the liver. Zhang *et al*. modified a gold nanocluster (GNC) with HIV‐1 trans‐activating transcriptor (TAT) peptide to combine with a Cas9‐encoding plasmid, and then were further coated with a galactose‐based liposome layer for liver targeting (**Figure** **12**A).^[^ ^105^ ^]^ The in vitro results reached 57% gene‐editing efficiency and showed corresponding reduced protein expression (Figure 12B), and the in vivo experiments also demonstrated a significant decrease of PSCK9 protein (Figure 12C) and reduced 30% serum level of low‐density lipoprotein cholesterol (LDL‐C) (Figure 12D).

Ping's group reported a photothermal‐responsive system sensitive to the NIR‐II wavelength, namely nanoCRISPR, composed of PSS/Au nanorod (APC) and a heat‐responsive HSP70 promoter‐driven Cas9 plasmid.^[^ ^106^ ^]^ For gene editing in the liver, the galactose‐mediated nanoCRISPR was used to treat Fas‐induced fulminant liver failure and showed 18% indel mutation rate in vitro and improved outcomes with reduced liver congestion. More recently, this group reported another PEI‐coated Au nanorod (AuNR) with a high aspect ratio (AR) to deliver CRISPR/Cas9 plasmids.^[^ ^107^ ^]^ They identified AuNR with high AR‐enabled excellent CRISPR delivery for Fas gene editing with 10.5% and 7.6% efficiency in vitro and in vivo, respectively, thus protecting mouse models from liver fibrosis. Lately, they changed the target into PD‐L1 gene for cancer immunotherapy, and the results demonstrated significant gene disruption and heat‐induced activation of immunogenic cell death (ICD).^[^ ^108^ ^]^

While for other inorganic nanoparticles, there gradually appeared mesoporous silicon material (MSN), graphene oxide, and black phosphorus nanosheet (BP) are also as vectors for gene‐editing components.^[^ ^109^ ^]^ An example for HCC therapy is that Zhang *et al*. reported a PAMAM‐aptamer‐modified hollow MSN (HMSN) to co‐deliver sorafenib and CRISPR/Cas plasmid‐targeting EGFR (epidermal growth factor receptor) as a synergistic strategy for HCC inhibition.^[^ ^110^ ^]^ This delivery system showed efficient accumulation in the HCC tumor site, exerting over 60% editing efficiency without off‐target effect and releasing the side effects of sorafenib. It is increasingly clear that the new results with inorganic nanocarriers have provided insight into available choices for CRISPR‐based nanomedicine for viral hepatitis and HCC.

##### Cell‐Derived Nanoparticles

Cell‐derived vesicle‐based delivery systems, especially exosomes, are another emerging direction for non‐viral CRISPR delivery. As an essential participant in cell‐to‐cell signal and material transfer, vesicles are stable as well as less immunogenic, and can efficiently pass through some hard‐entering membranes. Additionally, cell‐derived membranes can be modified with some targeting ligands, ensuring the specificity and accuracy of delivery systems.^[^ ^111^ ^]^

**Figure 13:**
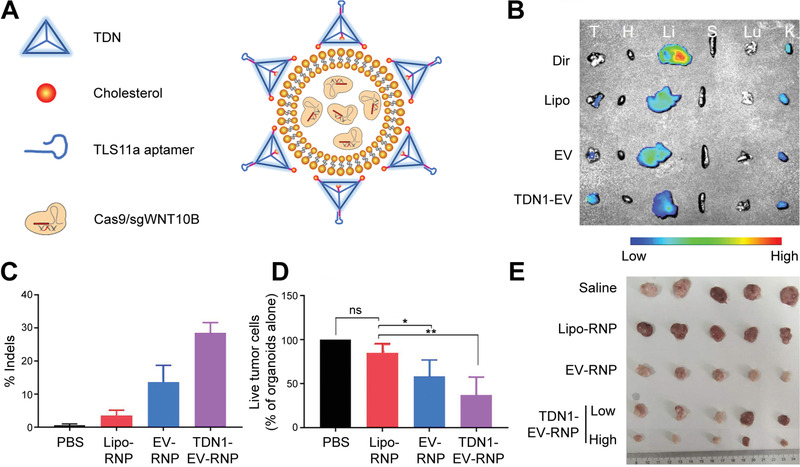
The HEK‐293T cell‐derived exosome was engineered with TLS11a aptamer‐modified TDNs to deliver CRISPR/Cas9 RNP for HCC therapy. A) Structure of TDN1‐EV loaded with CRISPR RNP targeting the WNT10B gene. B) Tumor‐targeting efficiency showed by fluorescent biodistribution image after 12 h of intravenous injection. T, tumor; H, heart; Li, liver; S, spleen; Lu, lung; K, kidney. C) The indel analysis of the HepG2 cells treated by different Cas9/sgWNT10B vectors. D) Quantification of organoid killing with different Cas9/sgWNT10B vectors. *p < 0.05, **p < 0.01, (one‐tailed *t*‐test). ns: *p* > 0.05. E) Representative images of xenograft tumors harvested from HCC mouse models. Reproduced with permission.^[^ ^112^ ^]^ Copyright 2020, Oxford University Press.

For anti‐HBV therapy, Chen *et al*. carried out endogenous exosomes to deliver the CRISPR/Cas9 system with HBV‐specific gRNA,^[^ ^83a^ ^]^ and the in vitro results indicated that this system could efficiently cut the HBV DNA transfected into Huh7 cells. For HCC therapy, extracellular vesicles (EVs) derived from cancer cells and modified on the surface have been investigated for targeted delivery of CRISPR components. The representative example is a recent study of the HEK‐293T cell‐derived exosome decorated with aptamer‐conjugated tetrahedral DNA nanostructures (TDNs) to deliver CRISPR cargos (**Figure** **13**A).^[^ ^112^ ^]^ With the decoration of selected TDN1 (one TLS11a aptamer and three cholesterol anchors), the engineered exosome packaged the CRISPR RNP‐targeting WNT10B and enabled specific location in the tumor sites (Figure 13B). The antitumor effects were verified by about 30% gene‐editing efficiency in vitro (Figure 13C) and <50% tumor cell viability ex vivo (Figure 13D). Besides, intravenous injection of TLS11a aptamer‐modified exosome loaded with Cas9/sgRNA showed efficient editing and fivefold tumor growth inhibition than plain exosomes in the mouse model of HCC (Figure 13E).

##### Peptide/Protein‐Based Nanoparticles

Various proteins and peptides have become attractive delivery systems for CRISPR cargos with their advantages of easy conjugation, biocompatibility, and biodegradability.^[^ ^113^ ^]^ The positive charges and surface modifications of protein/peptide‐based systems can condense CRISPR components and improve delivery and gene transfection.^[^ ^114^ ^]^ Therefore, the in vitro and in vivo gene editing in the liver can be facilitated by peptide/protein‐based methods.

The cell‐penetrating peptides (CPPs) with short peptide sequences can go through the cell membrane and chemically modify with CRISPR cargos.^[^ ^88c^ ^]^ Recently, a tandem peptide‐based nanocomplex, CRISPR‐GPS (guiding peptide sequences), was used to delivered RNP for gene editing in multiple cell lines.^[^ ^115^ ^]^ The tandem peptide lipid was composed of a cell‐targeting peptide head, a CPP, and a lipid tail. The demonstrated CRISPR‐GPS system could be further developed to target hepatic cells with a change into liver‐targeting peptide.

##### Viral Vectors and Non‐Viral Nanoparticles: Status and Prospects

Viral and non‐viral vectors are the two main streams as CRISPR delivery platforms for the next‐generation gene therapy, and the field of non‐viral CRISPR delivery is developing exponentially (**Table** **7**). Viral vectors, for example, AAVs, are common carriers in clinics.^[^ ^31b^ ^]^ In contrast, non‐viral vectors show lower immunogenicity, minimal genomic integration, flexible universality, and lower production cost.^[^ ^4^, ^54^, ^88^ ^]^ In the field of CRISPR/Cas‐based nanomedicine, though viral vectors are common and conventional carriers for gene delivery, they have gradually faced issues, such as tissue‐specific tropism that limits the targeting diversity, immunogenicity, and tumorigenic dangers due to random genome insertion.^[^ ^116^ ^]^ A recent study indicated that the 10‐year‐long AAV gene therapy in dogs showed unexpected gene integrations and may induce cancer.^[^ ^117^ ^]^

**Table 7:** Comparison of viral and non‐viral vectors as CRISPR delivery systems

Delivery methods		Characteristics	Advantages	Disadvantages	Applications	Ref.
Viral vectors	Adenovirus	Small, simple, episomal, and broad host range	‐ High delivery efficiency ‐ Low off‐target effects	‐ Strong immunoreactivity ‐ Limited to a small producing scale	Transient Cas9 protein expression requirements in gene therapy	^[^ ^62^, ^142^, ^175^ ^]^
	AAV	Small, simple, relatively safe	‐ High delivery efficiency ‐ Rare immune responses	‐ Low loading capacity (<5kb), ‐Non‐specific tropism ‐ High cost ‐ Limited to a small producing scale	‐ Gene therapy ‐ Modeling of genetic diseases	^[^ ^15^, ^176^ ^]^
	Retrovirus (mainly, lentivirus)	Replication via reverse transcription; broad host range	‐ Stable expression ‐ High delivery efficiency ‐ High loading capacity	‐ Random integration causing insertional mutagenesis, oncogene activation ‐ Not suitable for in vivo experiments	Gene therapy for cancer, genetic diseases, and so on	^[^ ^58^, ^177^ ^]^
Non‐viral vectors	Liposome or lipid‐based NPs	Easy and suitable customization to be hepatocyte‐specific	‐ Low immune responses ‐ Easy synthesis and modification ‐ Low cost	‐ Low delivery efficiency	Gene therapy	^[^ ^175^ ^]^
	Polymer NPs	Tunable chemical and physical properties	‐ High loading capacity ‐ Low immune responses ‐ High stability ‐ Programmable	‐ Low delivery efficiency ‐ Unclear long‐time toxicity	Gene therapy	^[^ ^178^ ^]^
	Inorganic NPs	Easy surface modification	‐ High loading capacity ‐ Easy synthesis and modification ‐ High cell uptake	‐ Unclear long‐time toxicity ‐ High‐dose toxicity	Gene therapy	^[^ ^58^ ^]^
	Cell‐derived NPs	Direct derivation from cells	‐ High loading capacity ‐ Non‐immunogenicity ‐ Easy synthesis and modification ‐ Biocompatibility	‐ Low delivery efficiency ‐ Difficult for clinical use	Gene therapy	^[^ ^179^ ^]^
	Peptide/protein‐based NPs	Small size, controllable construction	‐ Easy synthesis and modification ‐ High cell uptake	‐ Unclear long‐time toxicity	Gene therapy	^[^ ^62^ ^]^

In the recent years, non‐viral nanomaterials have shown great potential for precise and controllable delivery of CRISPR. The non‐viral CRISPR nanocarriers have a great prospect when measures are taken to optimize the bio‐safety, efficiency, and targeting ability. First, considerations of safety include biocompatibility and low cytotoxicity.^[^ ^118^ ^]^ Second, the improved efficiency benefits from stable encapsulation and shielding, modified ligands, increased cellular and nuclear entry.^[^ ^58^, ^88^, ^119^ ^]^ Third, the targeting ability highlights the individual designs, such as targeting ligands and responsibility to external conditions.^[^ ^88^, ^119^ ^]^

More importantly, recent studies with non‐viral delivery systems have achieved effective genome editing in vivo, particularly in the liver,^[^ ^51c^ ^]^ but further improvement for optimizing in vivo therapeutic applications of viral hepatitis and HCC should be developed.

#### 4.2.3 Combination of Viral and Non‐Viral Vectors

**Figure 14:**
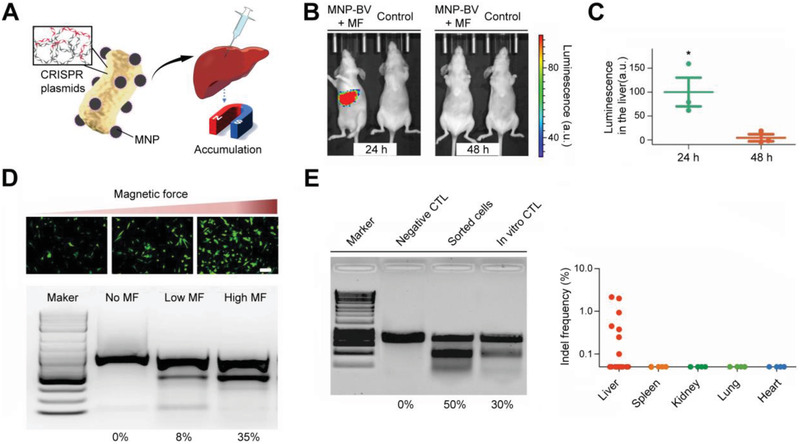
A complex of magnetic nanoparticle with recombinant baculoviral vector (MNP‐BV) for CRISPR delivery to achieve tissue‐specific gene editing. A) MNP‐BV loaded with CRISPR/Cas9 plasmid for liver‐targeting gene editing via extra magnetic field (MF). B) MF‐dependent gene editing in vitro. The targeting gene was vascular endothelial growth factor receptor 2 (VEGFR2). C) MF‐triggered liver‐specific accumulation of mice injected by MNP‐BV‐luciferase. *p < 0.05 (one‐tailed *t*‐test). D) Fluorescence image after transfection and T7E1 assay of VEGFR2 gene editing in vitro. Scale bar: 100 µm. E) T7E1 assay of VEGFR2 gene editing in vivo. CTL, control. Reproduced with permission.^[^ ^121^ ^]^ Copyright 2018, Springer Nature.

The combination of viral and non‐viral vectors may be beneficial by integrating the merits of both. For example, non‐viral delivery of CRISPR systems can give decent on‐target genome efficiency with minimal off‐target effects, whereas viral vectors deliver the sgRNA and the donor template with extremely high efficiency.^[^ ^10c^ ^]^ Qi *et al*. transfected Cas9 with lipofectamine and transduced gRNA via lentivirus in the HBV‐infected HepG2 cells.^[^ ^120^ ^]^ They used CRISPR/Cas9 to target the POLK gene for the suppression of rcDNA conversion, which significantly eliminated virus production. Moreover, Zhu *et al*. reported a comprehensive magnet‐sensitive transmission with a complex of magnetic nanoparticle and recombinant baculoviral vector (MNP‐BV) loaded with CRISPR/Cas plasmids (**Figure** **14**A).^[^ ^121^ ^]^ They applied the local presence of an MF to achieve spatial control of gene editing both in Hepa 1–6 cells (Figure 14B) and in the mouse liver (Figure 14C,D), achieving over 30% indel mutant rate. Promisingly, combinational strategies of viral and non‐viral CRISPR delivery are regarded as reliable strategies to treat liver diseases.

## 5 Potential CRISPR Therapeutics for Viral Hepatitis and Hepatocellular Carcinoma

### 5.1 Potential Key Molecules as CRISPR Therapeutic Targets

Moving forward, CRISPR/Cas editing to treat viral hepatitis majorly focuses on targeting HBV cccDNA and HCV RNA. Potentially, CRISPR/Cas9 could also be applied to target crucial host factors and cofactors in viral replication.^[^ ^122^ ^]^ Such key molecules under CRISPR‐mediated engineering may inhibit the infection of five types of hepatitis viruses and may act as alternatives for antiviral therapy in the liver.

For CRISPR‐mediated therapy, it is also necessary to discover liver cancer mutations of TSGs, oncogenes, and other tumor‐associated targets. Recently, approaches like CRISPR/Cas screening and disease models for validation have discovered a list of potential therapeutic targets of CRISPR‐based therapy in HCC (**Table** **8**). The emerging promising therapeutic targets with well‐designed CRISPR delivery systems can contribute to the bright future of nanomedicine.

**Table 8:** Potential therapeutic targets of CRISPR‐based therapy in HCC

Potential targets		Mechanisms	Ref.
Suppressor genes	Nf1	A tumor suppressor mutated in neurofibromatosis negative regulator of RAS signaling pathway with the downstream protein HMGA2	^[^ ^149^ ^]^
	AT‐rich interactive domain 2 (ARID2)	A component of the SWItch/Sucrose Non‐Fermentable (SWI/SNF) complex which frequent mutated in HCC	^[^ ^180^ ^]^
	PRDI‐BF1 and RIZ homology domain containing 8 (PRDM8)	Frequently downregulated in HCC and inhibiting PI3K/AKT/mTOR signaling pathway via regulating nucleosome assembly protein 1‐like 1 (NAP1L1)	^[^ ^181^ ^]^
	CDH1	Downregulated in HCC, encoding E‐cadherin for cell‐cell interaction and negatively regulating of EMT as well as metastasis	^[^ ^182^ ^]^
	Axis inhibition protein 1 (AXIN1)	A negative regulator of the Wnt/*β*‐Catenin cascade	^[^ ^183^ ^]^
Oncogenes	High‐affinity hexokinase (HK2)	Performing high‐affinity to HCC cells	^[^ ^87b^ ^]^
	Hepatic leukemia factor (HLF)	An oncofetal protein reactivated in HCC by SOX2 and OCT4	^[^ ^157^ ^]^
	Facilitates chromatin transcription (FACT)	A histone chaperone participating in DNA repair‐related and transcription‐related chromatin dynamics, essential to expeditious HCC oxidative stress response; synergistic antitumor effects of FACT inhibition and sorafenib	^[^ ^85a^ ^]^
	Transformation/transcription domain‐associated protein (TRRAP)	Promoting HCC cell proliferation by activating mitotic genes	^[^ ^184^ ^]^
	Cyclin‐dependent kinase 12 (CDK12)	Synergistic antitumor effects of CDK12 inhibition and sorafenib	^[^ ^85d^ ^]^

### 5.2 CRISPR Technology in CAR‐T Cell Therapy for Hepatocellular Carcinoma

Combining CRISPR/Cas9 and chimeric antigen receptor T (CAR‐T) cells, both revolutionary technologies, perform excellent prospects for enhanced therapeutic efficiency and safety in cancer immunotherapy.

CAR‐T cell therapy is one emerging area of cancer immunotherapy. The T cells, isolated from the patient body, are engineered with “GPS navigation” and “combat equipment” to rapidly recognize and kill cancer cells. Subsequently, these T cells are given back to the same patient.^[^ ^123^ ^]^ CAR‐T therapy has been developed into the third generation and has achieved remarkable efficacy in treating chronic myelogenous leukemia, acute myelogenous leukemia, non‐Hodgkin lymphoma, multiple myeloma, and some solid tumors.^[^ ^123^, ^124^ ^]^

**Figure 15:**
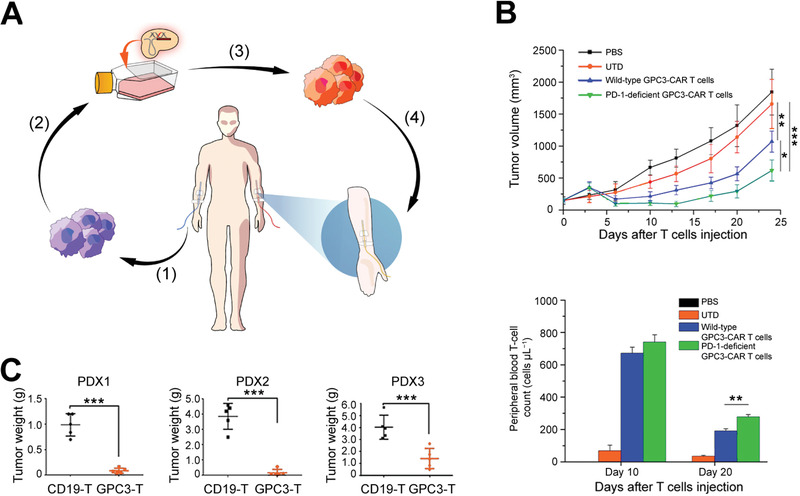
Integration of CAR T therapy and CRISPR/Cas technology. A) Description of combined CAR T and CRISPR therapy. (1) Isolation of T cells from the patient; (2) CRISPR‐mediated engineering in T cells; (3) amplification of edited T cells; (4) re‐infusion of edited T cells into the patient. B) Established PD‐1 disable GPC3‐CAR T cells via CRISPR/Cas technology to treat HCC. *p < 0.05, **p < 0.01, ***p < 0.001 (two‐tailed *t*‐test). Reproduced with permission.^[^ ^125^ ^]^ Copyright 2018, Frontiers. C) CRISPR‐edited GPC3‐CAR T cells to treat HCC in patient‐derived xenograft (PDX). ***p < 0.001 (two‐tailed *t*‐test). Reproduced with permission.^[^ ^126^ ^]^ Copyright 2017, Frontiers.

CRISPR/Cas technology could advance CAR‐T immunotherapy by enhancing efficiency, alleviating toxicity, and saving cost to perform super‐additive “1 + 1 > 2” effects (**Figure** **15**A). CAR T‐cell therapy has been synergized with CRISPR/Cas editing for active *ex vivo* trials.^[^ ^26^, ^30^ ^]^ Recently, Guo *et al*. used CRISPR/Cas technology to knock out immune checkpoints for HCC therapy (Figure 15B).^[^ ^125^ ^]^ They achieved CRISPR editing at the PD‐1 gene and thus improved the immunotherapeutic effects of CAR‐T cells. Besides, Jiang *et al*. produced the CRISPR‐edited CAR‐T cells targeting glypican‐3 (GPC3), the protein highly expressed in most liver cancers (Figure 15C).^[^ ^126^ ^]^ This strategy showed specific killing activity against the GPC3^+^ HCC cells in vitro and in vivo. Furthermore, Liu and co‐workers used CAR T cells with antigen modification to target the AFP gene in AFP^+^ liver cancer cells.^[^ ^127^ ^]^ The results showed obvious tumor growth suppression in vivo and provided a targeting strategy for CRISPR‐based applications.

CAR T‐cell therapy combined with CRISPR can promote HCC therapy. Introducing a CRISPR/Cas system targeting immune‐related genes allows the engineered CAR T cells for better killing activities.^[^ ^128^ ^]^ However, CAR‐T therapy for solid tumors is generally harder than hemopoietic cancers due to the tumor heterogeneity,^[^ ^129^ ^]^ which becomes a significant obstacle for this combined strategy for HCC therapy.

### 5.3 Rational Designs of Better CRISPR Therapeutics

**Figure 16:**
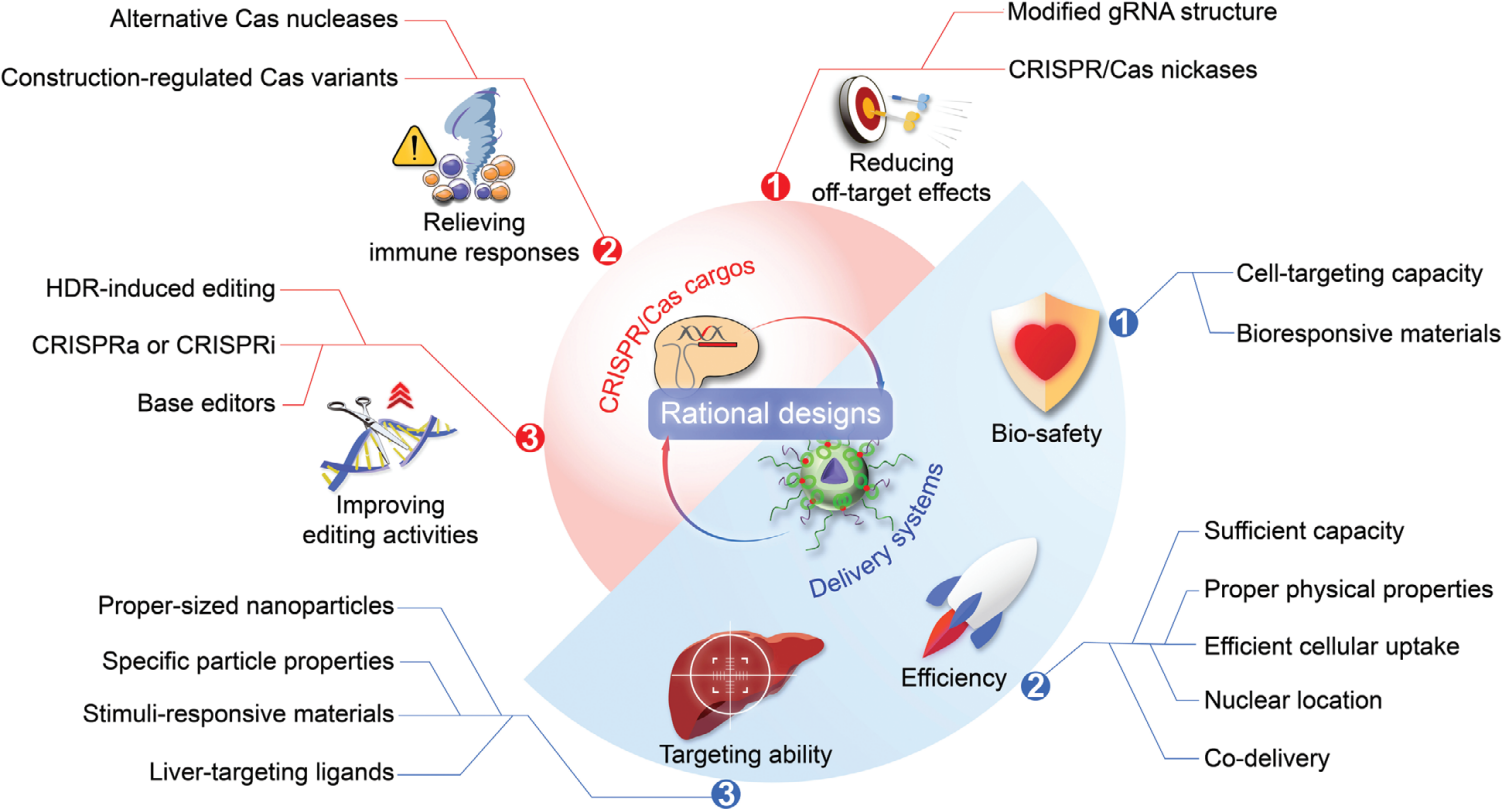
Rational designs of CRISPR therapeutics with cargos and delivery systems. The better CRISPR therapeutics can be improved by both well‐designed CRISPR/Cas cargos and safe, efficient, and targeted delivery vehicles.

To advance CRISPR/Cas‐mediated nanomedicine in the liver, immunogenicity and other adverse effects from CRISPR/Cas cargos or delivery vehicles should be solved. A rational design of CRISPR gene therapy to enhance the advantages as well as minimize side effects is necessary. In summary, successful CRISPR therapeutics require both a well‐designed CRISPR/Cas cargo and a safe, efficient and targeted delivery vehicle to go across extracellular and intracellular environments and completely execute therapeutic editing at the targeted site (**Figure** **16**).

#### 5.3.1 Designing Tips for CRISPR/Cas Cargos

A rational design for CRISPR/Cas cargo is the precondition of therapeutic editing. Selecting CRISPR/Cas tools and their delivery formats are the major factors that impact editing efficiency and in vivo delivery. First, the potential off‐target sites of CRISPR/Cas editing should be carefully assessed. Modifying the gRNA structure is a strategy to minimize off‐target severity that may cause tumorigenesis.^[^ ^51^, ^130^ ^]^ Besides, applying nCas9 with single strand cutting can enhance the editing specificity and potentially reduce undesirable indel mutations. Second, relieving immune responses can be realized by choosing alternative versions and construction‐regulated mutations of Cas endonucleases or fused with other functional agents.^[^ ^55^, ^131^ ^]^ Third, editing activities should be designed according to associated targets, disease state, and the physical properties of the liver. It has previously been said that the NHEJ pathway can be used for gene knockout with indels, while HDR‐induced editing tends to generate precise modification at a target locus. The HDR‐based pathway is encouraged when accurate gene correction is needed, and a repair template is required for this scenario. Next, CRISPRa or CRISPRi, without changes to the genome, is suitable for treating associated diseases by activating or repressing gene expression. Additionally, base editors can repair point mutations in genomic DNA without generating DSBs. Finally, the dose of CRISPR cargos should be sufficient to achieve therapeutic effects. Though the editing requirements for different diseases vary, ideal therapeutic outcomes generally come with high‐enough gene‐editing efficiency.

Furthermore, choosing the form of CRISPR/Cas tools for delivery should be careful. Since long‐term genetic modifications and Cas nuclease existence significantly increase off‐target cases, reducing the interaction duration between the Cas protein and the target DNA can safely decrease off‐target effects.^[^ ^51^, ^88^ ^]^ It thus prefers RNA or RNP delivery to avoid adverse gene integration, thus improving the editing specificity and reducing the off‐target potentials.^[^ ^13^, ^15^, ^34^, ^58^ ^]^ Alternatively, the replacement of a liver‐specific promoter in the CRISPR/Cas plasmid can realize selective CRISPR expression. Some cell‐specific or signal‐specific (such as drug‐, light‐ or other‐responsive) promoters have been studied before,^[^ ^106^, ^132^ ^]^ so equipping promoters into CRISPR plasmids is a practical way to control the editing expression.

#### 5.3.2 Designing Tips for Delivery Systems

Delivery systems for CRISPR/Cas cargos can ensure the ultimate success of therapeutic applications by balancing the benefits and risks of in vivo editing. Among the in vivo routes for nanoparticles loading with CRISPR/Cas components, the whole delivery system should first reduce unwanted clearance and degradation in the extracellular environment. Here, PEGylation is one of the most shielding methods for enhanced biocompatibility and prolonged body circulation.^[^ ^133^ ^]^ Moreover, directed liver‐targeting is the next goal to guarantee efficient gene delivery. Considering physiological barriers and molecular properties of hepatocytes, various target ligands and responsive materials sensitive to GSH, pH, magnetic field and so on, have been studied for this purpose.^[^ ^89^, ^116^, ^134^ ^]^ After the arrival in the liver, CRISPR activation needs entry into the cell membrane, endosomal escape, and location into cytosol or nucleus. In general, delivery strategies should be considered with their safety, efficiency, and targeting ability.

First, for safety, delivery vehicles should be biocompatible, minimize the immune harm and optimize the biodistribution in the body.^[^ ^51^, ^130^ ^]^ Recently, delivery vehicles with bioresponsive elements have attracted research attention due to the on‐time release of the CRISPR cargos and the biodegradability of the stimuli‐responsive materials.^[^ ^4^, ^133^ ^]^ Besides, cell‐targeting capacity, delivering to target lesions without impacting other normal parts, also decreases potential safety risks, which will be discussed as the following strategy.

Second, for efficacy, nanoparticles for delivery need enough capacity to load relatively large CRISPR elements (such as the plasmid form over 10 000 bp in length), and organizing a core–shell structure for inner packaging may benefit to protect the CRISPR cargo as stable encapsulation.^[^ ^119^, ^133^ ^]^ The physical properties, such as size and zeta potential, and the release kinetics of the CRISPR/Cas elements also need to be optimized for improved delivery efficacy.^[^ ^135^ ^]^ Next, approaches for endosomal escape, including lipid‐mediated membrane fusion, PEI‐mediated osmotic pressure, cationic polymer‐mediated swelling, and membrane destabilization, can facilitate the release of complete CRISPR cargos.^[^ ^95^ ^]^ Besides, nuclear localization sequences (NLSs) can stimulate signals for transport into the nucleus to fit the requirement that Cas9 reach the cell nucleus.^[^ ^136^ ^]^ For example, Rouet *et al*. fused Cas9 with three NLS tags showed double increased indels in HepG2 cells.^[^ ^137^ ^]^ Finally, co‐delivery systems for both CRISPR cargos and other therapeutic agents (*e.g*., photosensitizers^[^ ^138^ ^]^ and sorafenib^[^ ^89^, ^110^, ^133^ ^]^) can synergistically improve therapeutic efficacy.

Third, for targeting ability, liver targeting can be passive and active, namely decorating the delivery vehicles with targeting ligands and responsive materials to externally specific signals are the general strategies. In general, the liver is a unique organ that can rapidly passively accumulate systematically injected nanoparticles. Mainly, nanoparticles with a diameter <100 nm would selectively distribute toward hepatocytes.^[^ ^139^ ^]^ Besides the particle properties, the injection route also has effects, so taking a standard applying route‐like intravenous injection can improve passive liver targeting. In terms of active liver‐targeting, CRISPR/Cas delivery systems can locate and functionalize in the liver with the help of stimuli‐responsive materials and certain types of chemical or biological ligands. A given stimulus to control spatial and temporal delivery has received lots of attention.^[^ ^140^ ^]^ Stimuli‐responsive nanoparticles can facilitate endosome escape and liberate the CRISPR cargo with remote control. Moreover, designing ligands for ASGPR‐mediated hepatocyte targeting has been investigated most in the relative applications. A PEI‐modified gold nanorod for CRISPR delivery achieved triple‐targeting capacity via employing TAT (a typical CPP), NLS, and galactose (a ligand for ASGP receptor‐expressing hepatocytes).^[^ ^107^ ^]^ The galactose‐modified system was used for PCSK9 gene knockout in the liver and disrupted the target gene and relative protein expression. Another liver‐targeting example conjugated triantennary *N*‐acetylgalactosamine to a CRISPR RNP with a disulfide bond. The resulting trimeric complex showed higher cell uptake and editing efficiency in HepG2 cells than unmodified RNP.

## 6 Perspectives and Future Directions

CRISPR/Cas technology is a practical strategy for gene manipulation, including gene knockout, knockin, regulations, modifications, imaging, and base substitution. Although there are still concerns on off‐target effects and immunogenicity, it is promising to advance the nanotheranostics of liver diseases. In the past few years, studies on the CRISPR/Cas system have achieved remarkable results in hepatology.^[^ ^32^, ^141^ ^]^ Focusing on viral hepatitis and HCC, there has been emerging research for the CRISPR‐based application for diagnostics and therapeutics.^[^ ^81^, ^142^ ^]^

On one hand, CRISPR‐Dx for detecting viral hepatitis and HCC has tremendous potential for emerging diagnostic markers and developing CRISPR‐based detectors. As more disease‐related molecules have been discovered, significant considerations should be taken to choose suitable markers that specifically indicate a defined diagnosis. At the same time, CRISPR‐Dx as a disruptive innovation can enhance general performance and practicality. The assessments of performance include sensitivity and accuracy, robustness, signal transformation and quantitation, and multiplexing capability. The improved practicality needs: 1) free from equipment, 2) ease and portability of use, 3) broad applicability, and 4) rapid speed of assay.

On the other hand, recent years have witnessed considerable expansion of CRISPR/Cas toolbox by exploring the therapeutic targets and delivery vectors. We anticipate that the nanotechnology‐based delivery of CRISPR/Cas can serve as powerful therapeutic genome editing strategy for viral hepatitis and HCC treatment. From the clinical point of view, detailed accounts of both therapeutic targets and delivery systems are critical. Targets had better be focused on the specific lesion sites, for which CRISPR/Cas therapeutic editing can be carried out without damaging non‐target organs or cells. To achieve enhanced efficacy, multiple targets might be in need. Importantly, a safe, efficient, and specific delivery system ensures practical CRISPR/Cas editing at various targets. Safety requires mainly the transient CRISPR/Cas expression and the nanoparticles with good biocompatibility as well as sufficient encapsulation capacity. The efficiency can be improved by optimizing the CRISPR/Cas itself, implementing delivering measures of rapid endosome escape and nucleus location. Finally and most significantly, rational strategies for specifically targeting hepatocytes can be divided into two parts. For one thing, the Cas9 protein can be driven via a liver‐specific promoter. In this regard, several excellent papers reported tissue‐specific promoters to trigger CRISPR/Cas9 activity with specificity and accuracy.^[^ ^106^, ^132^ ^]^ Nanoparticles can be also engineered with externally controlled spatially and temporally as well as active ligand‐mediated targeting. Targeted delivery vehicles can be activated locally with different pH environments and additional optical, thermal, or magnetic fields. In another way, currently applied liver‐associated ligands are HBV‐related pre‐S1 peptide,^[^ ^134^, ^143^ ^]^ lactose, or galactose (Gal) for targeting to ASGPR,^[^ ^89^, ^104^, ^107^ ^]^ transferrin receptors,^[^ ^144^ ^]^ and so forth.

In the future, the next‐generation CRISPR/Cas‐based systems will hopefully advance this exciting area of diagnostics and therapeutics for liver‐associated diseases, especially viral hepatitis and HCC. Since there are also significant challenges that hinder translating the CRISPR/Cas technology into accurate diagnostics and therapeutics, how to find the balance between editing efficacy and potential side effects, mainly off‐target effects and immunogenicity, is the key. With emerging markers and targets discovered, approaches for CRISPR‐associated designing and delivery will take great efforts to push forward the theranostics of viral hepatitis and HCC.

## Conflict of Interest

The authors declare no conflict of interest.
